# ABA triblock copolymers prepared with poly(ε-caprolactone) and PEG, PTHF, and PPG macroinitiators as the central segment: synthesis, characterization, and thermal properties

**DOI:** 10.1039/d5ra06419h

**Published:** 2025-11-07

**Authors:** Miriam P. Barrera-Nava, Gerardo González García, José Bonilla Cruz, Kenneth J. Shea, José E. Báez

**Affiliations:** a Department of Chemistry, University of Guanajuato (UG) Noria Alta S/N 36050 Guanajuato Mexico jebaez@ugto.mx; b Advanced Functional Materials & Nanotechnology Group, Centro de Investigación en Materiales Avanzados S. C. (CIMAV-Unidad Monterrey) Av. Alianza Norte 202, Autopista Monterrey-Aeropuerto Km 10, PIIT Apodaca-Nuevo León 66628 C.P. Mexico; c Department of Chemistry, University of California, Irvine (UCI) California 92697-2025 USA

## Abstract

A total of 36 ABA-type triblock copolymers were synthesized *via* ring-opening polymerization (ROP) of ε-caprolactone (CL) using three different macroinitiators as segment B: poly(ethylene glycol) (PEG), poly(tetrahydrofuran) (PTHF), and poly(propylene glycol) (PPG). A range of molecular weights (*M*_n_ = 200–1000 g mol^−1^) and degrees of polymerization (DP) were used for segment A (DP = 5, 10, 15, and 20). MALDI-TOF analysis of the triblock copolymers revealed complexity in the pattern of the molecular weight distribution, where each DP of segment A (PCL) corresponded to a specific molecular weight distribution of segment B. Additionally, end group analysis indicated an α,ω-hydroxytelechelic species, which was supported by MALDI-TOF and ^1^H NMR studies. Increases in the DP of PCL segments resulted in higher melting temperatures (*T*_m_) and enthalpies of fusion (Δ*H*_m_), suggesting that crystallinity increases in a proportional manner. The influence of the B segment on the PCL segment was evidenced by a decrease of enthalpy and thus crystallinity, which was attributed to the disruption of the polyether segment generating amorphous domains in the PCL segment. Poly(ester urethane)s (PEUs) derived from ABA triblock copolymers showed plastic behavior with relatively poor mechanical properties.

## Abstract 2

Three different families of triblock copolymers ABA derived from PCL and polyethers such as PEG, PTHF, and PPG were studied in a systematic manner.

## Graphical abstract

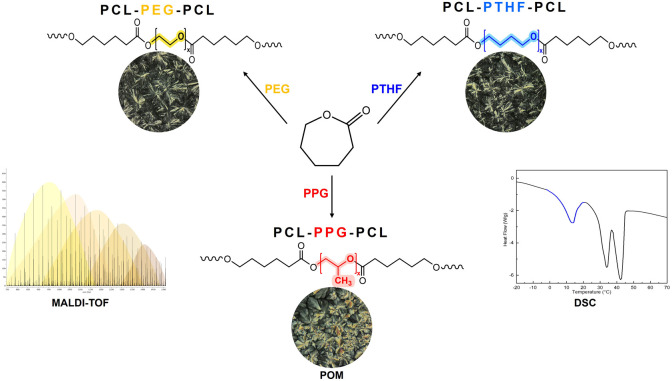

## 1 Introduction

Aliphatic polyesters are the most extensively studied class of biodegradable and biocompatible polymers. One example is poly(ε-caprolactone) (PCL), a hydrophobic polyester with a low melting point, high solubility, and an extraordinary ability to form blends. ε-caprolactone and its copolymers have been extensively used as drug-delivery carriers and tissue-engineering scaffolds due to their tissue compatibility, nontoxicity, and good biodegradability.^1–6^ PCL is prepared by ring opening polymerization (ROP) of ε-caprolactone using a metallic catalyst (most commonly stannous octanoate) and an initiator.^7–9^ It shows high-molecular-chain flexibility, outstanding processability, and a high degradation rate that can be manipulated by changing the molecular weight, crystallinity, or structure of PCL by inserting other groups.^10,11^

One of the techniques to modify the structure of PCL involves the formation of block copolymers. Of interest are triblock copolymers derived from PCL in combination with another polymer to provide a hydrophobic/hydrophilic balance. This allows for an intrinsic amphiphilic environment in the main chain. If PCL is copolymerized with poly(ethylene glycol) (PEG) to prepare block copolymers, their hydrophilicity and biodegradability can be increased, which is useful in a wide variety of applications.^12,13^ PEG is a hydrophilic polyether that is soluble in water and in organic solvents, as well as being nontoxic and biocompatible. Furthermore, it lacks antigenicity and immunogenicity, which allows it to be used for many clinical applications, such as synthetic dressing material for wound healing,^14^ tissue engineering,^15,16^ and blood substitutes.^17^ ABA-type triblock copolymers from PCL-PEG-PCL have been reported to have a higher gel modulus than other structures, which allows the formation of micelles in aqueous solutions^18,19^ and thermosensitive hydrogels^20,21^ for the delivery of drugs^22–24^ and proteins,^25^ 3D printing,^26^ and loaded copolymeric nanoparticle drug carriers.^27–30^

Other compounds can be used to synthesize triblock copolymers derived from PCL, such as poly(propylene glycol) (PPG) and polytetrahydrofuran (PTHF). The use of PPG in copolymers with PCL has been studied less than those with PEG/PCL, but PCL-PPG-PCL copolymers exhibit mechanical properties that are not shown by the individual homopolymers and appear to be promising for the development of interesting biomaterials.^31–33^ PPG is used in industry for the fabrication of lubricants, stabilizers, removers, chemical intermediates, and biomedical applications (mainly in the form of copolymers with PEG).^34,35^ The molecular structure of these triblock copolymers can make them form polymeric micelles in an aqueous medium, which may be useful as drug carriers. The hydrophobic PCL segments can form the inner core of the micelles, whereas the hydrophilic PPG segments can form the outer shell.^36,37^

Tetrahydrofuran can be subjected to cationic polymerization to obtain polytetrahydrofuran (PTHF), which is mainly used in the manufacture of elastomers and polyurethane resins.^38,39^ PTHF is used as a biocompatible soft segment in thermoplastic polyurethane (TPUs) elastomers^40,41^ due to its low glass-transition temperature (*T*_g_), excellent resiliency, high fungal resistance, and hydrolytic stability.^42^ Thus, they have been used for the synthesis of copolymers of different architectures.^43–46^

PCL-PTHF-PCL triblock copolymers are employed as polyurethane precursors for soft tissue engineering,^47–51^ water-vapor-permeable coatings,^52^ and complexes with cyclodextrins.^53^ Polyurethanes derived from triblock copolymers of PEG/PCL have applications in waterborne polyurethane dispersion and display better hydrolysis resistance, softness, and water vapor permeability.^54,55^ They could be used to design systems that could interact with pulmonary mucosa in order to prepare nanocarriers for lung cancer drug.^56^ And also be used in conjunction with other compounds to obtain biodegradable nanocomposites^57,58^ or enzymatically degradable polyurethanes.^59^

Miscibility is an important factor in blending polymers that are hardly miscible. Blending PCL and natural polymers results in phase-separation defects in polymer blending. Hence, the use of a compatibilizer is a crucial factor in PCL applications,^60^ and triblock copolymers derived from PCL can serve this purpose. For example, to improve adhesion in blends between PLA and PCL phases, a copolymer of PCL/PTHF has been used,^61^ while for blends of PCL-PLA-PEG, a triblock copolymer of PCL/PEG leads to a slight increase in surface hydrophilicity.^62^

A systematic study of ABA triblock copolymers with PCL as A segment allows the establishment of clear relationships between the molecular structure and their thermal and morphological properties. This is an area that has been limited sense in the existing reports. Table 1 presents a comprehensive review of the literature revealing a predominance of the research on PCL-PEG-PCL systems in the context of biomedical applications, with PCL-PTHF-PCL systems being less studied, and PCL-PPG-PCL systems have received comparatively limited exploration. In fact, this last systems are generally evaluated in combination with another polyether, and not independently with PCL, making it challenging to attribute their intrinsic effects of PPG. In contrast, other PEG-PPG-PTHF triblock systems have been explored primarily from the perspective of their capacity biomedical applications, where the emphasis is on hydrophilic compatibility and flexibility of the central chain.^63–65^ However, these triblocks copolymers have not been integrated within a unified experimental framework, limiting the evaluation of how each macroinitiator modulates PCL crystallinity (*x*_*i*_), thermal stability, or self-organization capacity.

**Table 1:** A comparative analysis of the applications of some copolymer systems

Copolymer	Application	Author and year
PCL-PEG-PCL	Core-shell micelles	Piao, 2003 ref. 12
	Tissue engineering applications derived from polyurethane (PU) nanocomposites	Noormohammadi, 2021 ref. 16
	Micelles as nanocarries for ocular delivery of dexamethasone	Alami-milani, 2018 ref. 24
	Doxorubicin (DOX)-loaded polymeric nanoparticles (NPs)	Zhang, 2016 ref. 29
	Drug-loaded with vancomycin	Singh, 2020 ref. 30
PCL-PTHF-PCL	PU for soft tissue engineering	Mi, 2017 ref. 43
	Segmented thermoplastic polyurethanes (STPU)	Rueda-Larraz, 2009 ref. 44
	Inclusion complexes (ICs) with cyclodextrins (CDs)	Li, 2004 ref. 48
	Electrospun nanofibers	Jiang, 2018 ref. 51
	Compatibilizer of immiscible PLA/PCL blends	Do Patrocínio, 2019 ref. 56
PCL-PPG-PCL	Biodegradable polyurethane scaffolds	Shi, 2025 ref. 33
	Micelles for potential drug carriers	Lee, 2011 ref. 36
PPG-PCL	ICs with α-cyclodextrin (α-CD)	Chung, 2007 ref. 34
PEG-PPG-PTHF	Injectable thermogels implants for long-term intraocular application	Zhang, 2022 ref. 63
PCL-PEG-PPG	Hydrogels for cell therapies	Brewer, 2020 ref. 64
PCL-PEG-PPG	Water-swelling of thermo-responsive poly(ester urethane)s	Loh, 2008 ref. 65

In order to comprehend the behavior of more complex triblock copolymers, such as PEG-PPG-PTHF systems, it is crucial to understand the influence of each polyether segment individually. This facilitates the identification of distinctive characteristics, such as the plasticizing effect of PPG or the phase segregation tendency of long PTHF chains.

**Scheme 1:**
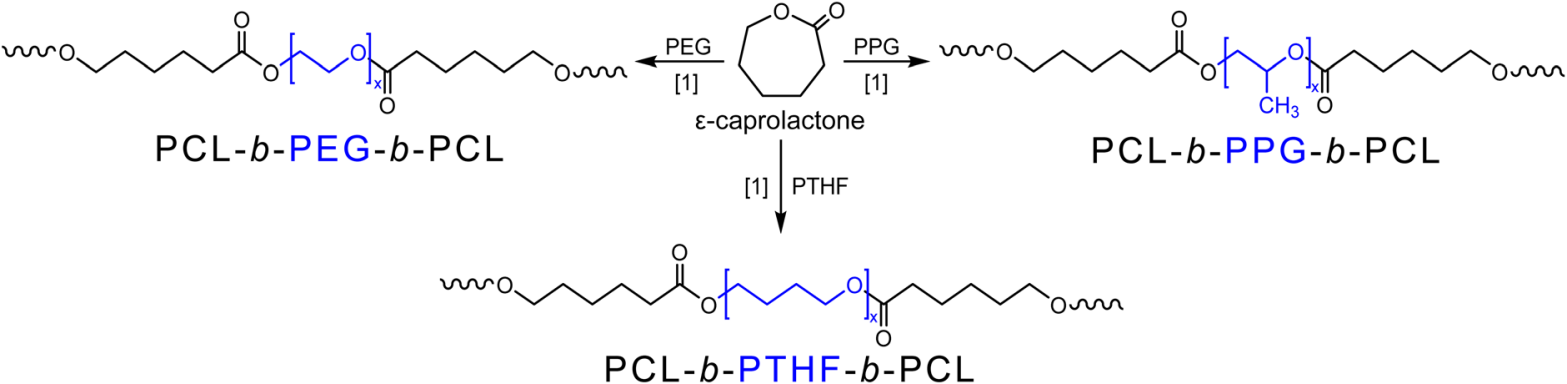
Ring opening polymerization (ROP) of ε-caprolactone (CL) to obtain triblock copolymers. Note [1] = 150 °C, 120 min, 3 mg Hep and four different DP_PCL_ = 5, 10, 15, and 20.

The motivation of this research is to study the effect of different types of polyether oligomers as the central segment B in triblock copolymers of the form ABA, where A is a poly(caprolactone) (PCL) segment. This work presents the synthesis, characterization, and comparison of a family of three species of triblock copolymers: HOPCL-*b*-PEG-*b*-PCLOH, HOPCL-*b*-PTHF-*b*-PCLOH, and HOPCL-*b*-PPG-*b*-PCLOH (Scheme 1). To achieve this, a systematic analysis of the growth of segment A and segment B was carried out to analyze the effect of block B on the PCL segment, to study the degree of polymerization, and to observe the impact that it has on the thermal properties of the triblock copolymer. To our knowledge, there has been no previous report of a systematic comparison between triblock copolymers derived from PCL and polyethers PEG, PTHF, and PPG. All polymeric species were characterized using different analytical techniques, such as ^1^H and ^13^C NMR, FT-IR, GPC, MALDI-TOF, POM, and DSC.

## 2 Experimental

### 2.1 Materials

Polyethylene glycol (PEG) (*M*_n_ = 200, 400, and 1000 g mol^−1^), poly(propylene glycol) (PPG) (*M*_n_ = 425, 725, and 1000 g mol^−1^), polytetrahydrofuran (PTHF) (*M*_n_ = 250, 650, and 1000 g mol^−1^) and ε-caprolactone (CL) were purchased from Aldrich Chemical Co. CL was dried over calcium hydride (CaH_2_) for 24 h and distilled under reduced pressure before use. Ammonium heptamolybdate tetrahydrate (NH_4_)_6_[Mo_7_O_24_]·4H_2_O (Hep) (Fluka) was ground with a pestle and mortar before use.

#### 2.1.1 Typical procedure for the synthesis of triblock copolymer poly(ε-caprolactone)-*b*-polyethylene glycol-*b*-poly(ε-caprolactone) (PCL-*b*-PEG-*b*-PCL)

Polymerization was performed in the absence of solvent (bulk polymerization) in a dried 25 mL round-bottom flask. Ammonium heptamolybdate tetrahydrate [(NH_4_)_6_[Mo_7_O_24_]·4H_2_O (Hep), 3 mg], ε-caprolactone (CL) (40 mmol), 4.56 g, and polyethylene glycol (PEG_400_) (*M*_n_ = 408 g mol^−1^) (4 mmol, 1.64 g) were charged and heated by stirring them in an oil bath at 150 °C for 2 h (molar ratio CL/Hep = 16 : 500 and CL/PEG = 10). Ammonium decamolybdate (NH_4_)_8_[Mo_10_O_34_] was obtained *in situ* in the solid state by thermal decomposition of ammonium heptamolybdate (NH_4_)_6_[Mo_7_O_24_].^8^

The product PCL-*b*-PEG_400_-*b*-PCL synthesized was analyzed without purification. Number-average molecular weight (*M*_n_) and conversion were monitored by ^1^H NMR. After reaction time, an aliquot of the crude of the reaction was dissolved in CDCl_3_ and derivatized with two drops of trifluoroacetic anhydride (TFAA) to prevent overlapping between methylene attached to hydroxyl and ethylene glycol groups and analyzed by ^1^H NMR.^66^ In ^1^H NMR spectrum, the peaks at 2.35 [–CH_2_–CO–O–, *I*_pol_, repetitive unit], 3.82 [F_3_C–CO–O–CH_2_–CH_2_–O–CH_2_–CH_2_–O–CO–, *I*_eg_, monosubstitution of polyethylene glycol], and 3.76 [–CO–O–CH_2_–CH_2_–O–CH_2_–CH_2_–O–CO–, *I*_eg_, bisubstitution of polyethylene glycol] were used to quantify the *M*_n_ in two steps:

(1) The degree of polymerization (DP). DP (NMR) = (*I*_pol_/#*H*_pol_ ÷ *I*_eg_/#*H*_eg_) + 2. The *I*_pol_ and *I*_eg_ represent the integrals of the methylenes obtained by ^1^H NMR from the polyester [–(CH_2_)_4_–CH_2_–CO–O–] and polyethylene glycol group [F_3_C–CO–O–CH_2_–CH_2_–(O–CH_2_–CH_2_)_*n*_–O–CO– and –CO–O–CH_2_–CH_2_–(O–CH_2_–CH_2_)_*n*_–O–CO–] peaks, respectively, #*H*_pol_ and #*H*_eg_ represent the number of protons that contributed to the peaks, the number 2 indicates the contribution of two end groups. Finally, the equation is DP(NMR) = (*I*_pol_/2 ÷ *I*_eg_/4) + 2. (2) The number-average molecular weight (*M*_n_). *M*_n_ (NMR) = [(MW (CL))·(DP (NMR))] + MW (macrodiol), where MW is the molecular weight of the repetitive unit (CL) and macrodiol (polyethylene glycol), respectively; DP (NMR) was previously calculated in step 1. Table 1 shows the average molecular weight (*M*_n_) obtained by ^1^H NMR, MALDI-TOF, and GPC, for the triblock copolymers derived from PEG, where the *M*_n_ calculated is similar to the values of *M*_n_ obtained by ^1^H NMR and MALDI-TOF.

*M*_n_ (calcd) = 1550, *M*_n_ (NMR) = 1680 (Conv. = 98%), *M*_n_ (GPC) = 2969, *M*_w_/*M*_n_ = 1.14, *M*_n_ (MALDI) = 1244. IR (cm^−1^) 3451 (*ν*, OH, PCL), 2941 (*ν*_as_, CH_2_, PCL), 2865 (*ν*_s_, CH_2_, PCL), 1722 (*ν*, C=O, PCL), 1471 (*δ*_s_, CH_2_, PCL), 1163 (*ν*_as_, C–(C=O)–O, PCL), 1044 (*ν*_as_, O–C–C, PCL), 732 (*ρ*, CH_2_, PCL). NMR data for PCL-*b*-PEG_400_-*b*-PCL (DP = 10). ^1^H NMR after derivatization with TFAA (500 MHz, CDCl_3_, ppm): *δ* 4.49 [F_3_C–CO–O–C**H**_2_–CH_2_–O–, PEG monosubstitution and unreacted PEG], 4.34 [–CO–CH_2_–CH_2_–CH_2_–CH_2_–C**H**_2_–O–CO–CF_3_, PCL], 4.25 [–CO–O–C**H**_2_–CH_2_–O–CH_2_–CH_2_–O–CO–, PEG bisubstitution and F_3_C–CO–O–CH_2_–CH_2_–O–C**H**_2_–CH_2_–O–CO–, PEG monosubstitution], 4.10 [(–CO–CH_2_–CH_2_–CH_2_–CH_2_–C**H**_2_–O–)_*n*_, PCL], 3.82 [F_3_C–CO–O–CH_2_–C**H**_2_–O, PEG monosubstitution and unreacted PEG], 3.71 [–CO–O–CH_2_–C**H**_2_–O–C**H**_2_–CH_2_–O–CO–PEG bisubstitution and F_3_C–CO–O–CH_2_–CH_2_–O–C**H**_2_–CH_2_–O–CO–, PEG monosubstitution], 2.36 [(–CO–C**H**_2_–CH_2_–CH_2_–CH_2_–CH_2_–O–)_*n*_, PCL], 1.76 [–CO–CH_2_–C**H**_2_–CH_2_–CH_2_–CH_2_–O–CO–CF_3_, PCL], 1.66 [(–CO–CH_2_–C**H**_2_–CH_2_–CH_2_–CH_2_–O–)_*n*_, PCL], 1.38 [(–CO–CH_2_–CH_2_–C**H**_2_–CH_2_–CH_2_–O–)_*n*_, PCL]. ^13^C NMR (125 MHz, CDCl_3_, ppm): …–[CH_2_^i^–CH_2_^i^–O]_*x*−1_–CH_2_^h^–CH_2_^g^–O–[C(=O)^f^–CH_2_^a^–CH_2_^b^–CH_2_^c^–CH_2_^d^–CH_2_^e^–O]_*n*–1_–C(=O)^f^–CH_2_^a^–CH_2_^b^–CH_2_^c^–CH_2_^d^–CH_2_^e′^–O–C(=O)^j^–CF_3_^k^: *δ* 174.25 (f), 157.79 (j), 114.18 (k), 70.25 (i), 69.09 (h), 67.83 (g), 64.56 (e′), 63.40 (e), 40.77 (j), 34.10 (a), 28.14 (d), 25.38 (b), 24.46 (c). Data characterization for PCL-*b*-PTHF_250_-*b*-PCL (DP = 10) and PCL-*b*-PPG_425_-*b*-PCL (DP = 10) are presented in SI.

#### 2.1.2 Synthesis of polyurethane (PEU) derived from triblock copolymer poly(ε-caprolactone)-*b*-polytetrahydrofuran-*b*-poly(ε-caprolactone) (PCL-*b*-PTHF_250_-*b*-PCL_10_) and hexamethylene diisocyanate (HDI)

The reaction was carried out in a 25 mL round-bottom flask previously dried, 1.5 g (1.084 mmol) of PCL-*b*-PTHF_250_-*b*-PCL_10_ [*M*_n_(NMR) = 1560] was added. Subsequently, 223 mg (1.322 mmol) of HDI [PCL-*b*-PTHF_250_-*b*-PCL_10_ : HDI molar ratio = 1 : 1.14] was added and dissolved in 11.28 mL of 1,2-dichloroethane (DCE) [0.176 molal with respect to PCL-*b*-PTHF_250_-*b*-PCL_10_],^67^ and tin(ii) 2-ethylhexanoate [Sn(Oct)_2_] was added as catalyst [1 wt%, 32 mg ∼ 3 drops]. Then, the flask was placed in an oil bath at 80 °C for 3 hours. After the reaction time, a PU-film was obtained by casting at room temperature on a leveled Teflon surface within a fume cupboard covered with a conical funnel to protect it from dust and allow a slow solvent evaporation for 12 h. After, the PU film was released and dried under a vacuum. Following the same methodology, a PU derived from PCL-*b*-PEG_200_-*b*-PCL_10_ and a PU derived from PCL-*b*-PPG_425_-*b*-PCL_10_ were synthesized.

IR (cm^−1^): 3389 (*ν*, N–H, urethane), 2942(*ν*_as_, CH_2_, PCL), 2864 (*ν*_*s*_, CH_2_, PCL), 1721 (*ν*, C=O, PCL), 1626 (*ν*, C=O, urethane), 1530 (*δ*, N–H, urethane), 1471 (*δ*_s_, CH_2_, PCL), 1186(*ν*_as_, C–(C=O)–O, PCL), 1045 (*ν*_as_, O–C–C, PCL), 732 (*ρ*, CH_2_, PCL). NMR data for PU-2_PTHF_ derived from PCL-*b*-PTHF_250_-*b*-PCL (DP = 10) and HDI. ^1^H NMR (400 MHz, CDCl_3_, ppm): *δ* 4.74 [–N**H**–, urethane], 4.04 [(–CO–CH_2_–CH_2_–CH_2_–CH_2_–C**H**_2_–O–)_*n*_, PCL], 3.62 [unreacted PTHF], 3.40 [–O–C**H**_2_–CH_2_–CH_2_–C**H**_2_–O, PTHF backbone], 3.12 [–CO–NH–C**H**_2_–(CH_2_)_4_–C**H**_2_–NH–CO–, urethane], 2.29 [(–CO–C**H**_2_–CH_2_–CH_2_–CH_2_–CH_2_–O–)_*n*_, PCL], 1.61 [–CO–CH_2_–C**H**_2_–CH_2_–C**H**_2_–CH_2_–O–CO–, PCL and –O–CH_2_–C**H**_2_–CH_2_–CH_2_–O–CO, PTHF], 1.47 [–CO–NH–CH_2_–C**H**_2_–CH_2_–CH_2_–C**H**_2_–CH_2_–NH–CO–, urethane], 1.36 [(–CO–CH_2_–CH_2_–C**H**_2_–CH_2_–CH_2_–O–)_*n*_, PCL and –CO–NH–CH_2_–CH_2_–C**H**_2_–CH_2_–CH_2_–C**H**_2_–NH–CO–, urethane]. ^13^C NMR (100 MHz, CDCl_3_, ppm): …–[CH_2_^k^–CH_2_^l^–CH_2_^l^–CH_2_^k^–O]_*x*–1_–CH_2_^j^–CH_2_^i^–CH_2_^h^–CH_2_^g^–O–[C(=O)^f^–CH_2_^a^–CH_2_^b^–CH_2_^c^–CH_2_^d^–CH_2_^e^–O]_*n*−1_–C(=O)^f^–CH_2_^a′^–CH_2_^b′^–CH_2_^c′^–CH_2_^d′^–CH_2_^e′^–O–C(=O)^m^–NH–CH_2_^n^–CH_2_^o^–CH_2_^o^–CH_2_^o^–CH_2_^o^–CH_2_^n^–NH…: *δ* 173.65 (f), 156.88 (j), 70.80 (k), 70.39 (j), 64.65 (e′), 64.25 (e), 62.68 (g), 40.90 (n), 34.22 (a), 32.43 (a′), 28.45 (o), 26.6 (c), 26.5 (c′), 26.40 (h), 26.36 (l), 26.33 (i), 25.63 (d), 25.41 (d′), 24.74 (b′), 24.68 (b) (Fig. S12).

### 2.2 Characterization methods

Nuclear magnetic resonance (NMR) ^1^H NMR was recorded at room temperature on Bruker Avance III HD 500 MHz spectrometer (500 MHz ^1^H and 125 MHz ^13^C). CDCl_3_ was used as a solvent, and all spectra were referenced to the residual solvent CDCl_3_ [*δ* (ppm) 7.26 (^1^H), 77.0 (^13^C)]. *Fourier transform infrared spectroscopy* (*FT-IR*): triblock copolymers were recorded with attenuated total reflectance spectroscopy (ATR) accessory in a PerkinElmer Spectrum One FT-IR spectrometer. *Differential scanning calorimetry* (*DSC*): thermograms were performed in a Mettler Toledo DSCQ2000 TA instrument. Three scans were obtained with two heating (25–100 °C and −30 to 100 °C) and one cooling (100 to −30 °C) between them, at a rate of 10 °C min^−1^ and under a nitrogen purge. The degree of crystallinity (*x*_PCL_) for PCL was calculated from the endothermic peak area (Δ*H*_m_) by *x*_PCL_ = Δ*H*_m_/Δ*H*_m_0__, where *H*_m_0__ is the heat of fusion for perfect PCL (135.3 J g^−1^)^68^ crystals. *Gel permeation chromatography* (*GPC*): GPC measurements were determined using an Agilent Technologies PL-GPC 220 Gel Permeation chromatograph, with PLgel 5um MIXED-D Columns to elute samples at the flow rate of 1 mL min^−1^ HPLC grade tetrahydrofuran (THF). Polystyrene standards (Polymer Laboratories) were used for calibration. Matrix-Assisted Laser Desorption Ionization Time-of Flight (MALDI-TOF) MALDI-TOF spectra were recorded in the reflector mode by using an AB SCIEX TOF/TOF 5800 SYSTEM equipped with a nitrogen laser emitting at *λ* = 349 nm, an input bandwidth = 1000 MHz with a 3 ns pulse width and working in positive mode and delayed extraction. A concentration of 10 mg mL^−1^ of 2,5-dihydroxybenzoic acid (DHB) in THF and water as solvents were used. Copolymer samples (3 mg mL^−1^) were dissolved in THF (at room temperature), and then 10 μL of sample solution was mixed with 10 μL of matrix solution (50/50, vol/vol) in a centrifuge tube (Eppendorf tube) and mixed in vortex. Different aliquots were placed on a stainless-steel plate, and the solvent was evaporated to make the film start the acquisition. *Polarized optical microscopy* (*POM*): POM micrographs were obtained using a Nikon light Eclipse E200 microscope, photographs were taken using iPhone 13 mini. Triblock copolymers were mounted on glass slides as a thin film melted at 80 °C and applying manual pressure between the two slides containing the sample and cover glass. The samples were cooled at room temperature before analysis. Samples were collected with a magnification of 10× and 40×. *Mechanical properties*: the mechanical properties of the poly(ester urethanes) (PEUs) were measured in a Zwick/Roell Z005 instrument equipped with a 5000 N load cell. Type 3 dumbbell test pieces (according to ISO 37) were cut from the films. A crosshead speed of 200 mm min^−1^ was used. At least three samples were evaluated for each PEU.

## 3 Results and discussion

### 3.1 Part 1. triblock copolymers (PCL-*b*-PEG-*b*-PCL, PCL-*b*-PTHF-*b*-PCL, and PCL-*b*-PPG-*b*-PCL)

A family of three different molecules with diverse number-average molecular weights (*M*_n_) were derived from ether group macrodiols (polydisperse organic macromolecule): polyethylene glycol (PEG) [HO(–CH_2_–CH_2_–O–)H with *M*_n_ = 200, 400 and 1000 g mol^−1^], polytetrahydrofuran (PTHF) [HO(–CH_2_–CH_2_–CH_2_–CH_2_–O–)H with *M*_n_ = 250, 650 and 1000 g mol^−1^], and polypropylene glycol (PPG) [HO(–CH_2_–CH(CH_3_)–O–)H with *M*_n_ = 425, 725 and 1000 g mol^−1^]. These molecules were used as initiators in the ring-opening polymerization (ROP) of ε-caprolactone (CL) using four different degrees of polymerization (DP) (DP = 5, 10, 15, and 20) with decamolybdate anion as a catalyst in bulk polymerization at 150 °C for 120 min (Scheme 1). After the reaction time, a good conversion of over 89% was obtained in the case of copolymers with PEG and the copolymers with PPG, and higher conversions over 96% were obtained in the case of PTHF copolymers (Table 2) (Tables S1–S3). The DP values calculated by end-group analysis (^1^H NMR) [DP(NMR)] were similar to the value of DP(calcd) obtained by monomer and initiator feed, which is evidence of polymerization control.

**Table 2:** Triblock copolymers (PCL-*b*-B-*b*-PCL) prepared using different polyethers as central segment (B = PEG, PTHF and PPG of different molecular weight) as initiators in the ROP of CL

Sample	Initiator	Ether (%)^a^^,^^b^	DP (calcd)^c^	DP (NMR)^b^^,^^d^	*M*_n_ (calcd)^e^	*M*_n_ (NMR)^b^^,^^f^	*M*_n_ (GPC)^g^	*M*_n_ (calcd)/*M*_n_ (GPC)	*M*_w_/*M*_n_^g^	Conv (%)
PEG_200_		—	—	4.5	200	214				
PCL-*b*-PEG_200_-*b*-PCL_5_	PEG_200_	28	5	4.9	780	770	1410	0.55	1.18	92
PCL-*b*-PEG_200_-*b*-PCL_10_	PEG_200_	14	10	11.5	1350	1530	3145	0.42	1.24	95
PCL-*b*-PEG_200_-*b*-PCL_15_	PEG_200_	11	15	14.3	1990	1850	3988	0.49	1.22	98
PCL-*b*-PEG_200_-*b*-PCL_20_	PEG_200_	8	20	21.0	2440	2610	5628	0.43	1.24	98
PTHF_250_		—	—	3.3	250	254				
PCL-*b*-PTHF_250_-*b*-PCL_5_	PTHF_250_	28	5	5.6	820	890	1601	0.51	1.19	96
PCL-*b*-PTHF_250_-*b*-PCL_10_	PTHF_250_	16	10	11.4	1390	1560	3273	0.42	1.23	97
PCL-*b*-PTHF_250_-*b*-PCL_15_	PTHF_250_	12	15	16.2	1960	2100	4853	0.40	1.16	99
PCL-*b*-PTHF_250_-*b*-PCL_20_	PTHF_250_	10	20	20.3	2530	2570	5483	0.46	1.40	99
PPG_425_		—	—	6.8	425	396				
PCL-*b*-PPG_425_-*b*-PCL_5_	PPG_425_	43	5	7.8	1000	1280	2373	0.42	1.15	89
PCL-*b*-PPG_425_-*b*-PCL_10_	PPG_425_	27	10	14.2	1570	2020	4102	0.38	1.16	96
PCL-*b*-PPG_425_-*b*-PCL_15_	PPG_425_	20	15	18.7	2130	2530	6079	0.35	1.14	98
PCL-*b*-PPG_425_-*b*-PCL_20_	PPG_425_	16	20	22.7	2680	2980	5719	0.46	1.48	98

a Obtained from the equation % ether = (MW_initiator_/*M*_n_(NMR)) × 100; where MW_initiator_ is the molecular weight of initiator (HOPEGOH, HOPTHFOH, or HOPPGOH).

b Determined by ^1^H NMR in CDCl_3_.

c Obtained from CL/polyether feed molar ratio.

d Using end-group analysis by ^1^H NMR.

e Obtained from the equation *M*_n_(calcd) = (MW(CL))·(mmol CL/mmol polyether) + MW(polyether), where MW is the molecular weight of ε-caprolactone (CL, 114 g mol^−1^) monomer or initiator (HOPEGOH, HOPTHFOH, or HOPPGOH).

f Obtained from the equation *M*_n_(NMR) = (DP(NMR) × MW(repetitive unit)) + MW(polyether), where MW is the molecular weight of the repetitive unit (114 g mol^−1^) or initiator (HOPEGOH, HOPTHFOH, or HOPPGOH).

g Determined by gel permeation chromatography (GPC) using polystyrene standards.

Good concordance was observed between *M*_n_ (calcd) and *M*_n_ (NMR) for all triblock copolymers, which implied control of the polymerization. On the other hand, *M*_n_ (calcd) and *M*_n_ (GPC) showed significant differences (Table 2), with *M*_n_ (GPC) being approximately double the value of *M*_n_ (calcd) and higher than that *M*_n_ (NMR). This is relatively common due to the polystyrene standards used in the calibration curve. The polydispersity was moderate (1.09–1.53). For PCL, MacLain and Drysdale discovered that the *M*_n_ (calcd)/*M*_n_ (GPC) ratio is approximately 0.45.^69^ The *M*_n_ (calcd)/*M*_n_ (GPC) ratio is presented for the samples in Table 3, with values of *M*_n_ overestimation with a range from 0.35 to 0.55. Some GPC chromatograms are presented in SI (Fig. S1 and S2).

**Table 3:** Thermal properties of triblock copolymers (PCL-*b*-B-*b*-PCL) prepared using different polyethers as central segment (B = PEG, PTHF and PPG of different molecular weight) as initiators in the ROP of CL

Sample	Initiator	Ether (%)^a^^,^^b^	DP (calcd)^c^	DP (NMR)^b^^,^^d^	*M*_n_ (calcd)^e^	*M*_n_ (NMR)^b^^,^^f^	*T*_c_ (°C)^g^	Δ*H*_c_ (J g^−1^)^g^	*T*_mPCL_ (°C)^h^	Δ*H*_m_ (J g^−1^)^h^	*x*_*i*_ (%)^h^^,^^i^
PEG_200_		—	—	4.5	200	214			−50^j^	—	—
PCL-*b*-PEG_200_-*b*-PCL_5_	PEG_200_	28	5	4.9	780	770	—	—	6	0.7	0.5
PCL-*b*-PEG_200_-*b*-PCL_10_	PEG_200_	14	10	11.5	1350	1530	15	50	32,41	50	37
PCL-*b*-PEG_200_-*b*-PCL_15_	PEG_200_	11	15	14.3	1990	1850	20	73	35,42	76	56
PCL-*b*-PEG_200_-*b*-PCL_20_	PEG_200_	8	20	21.0	2440	2610	23	75	43,48	77	57
PTHF_250_		—	—	3.3	250	254			−16	53	
PCL-*b*-PTHF_250_-*b*-PCL_5_	PTHF_250_	28	5	5.6	820	890	−18	42	−16^k^,5,13	43(31^m^)	23
PCL-*b*-PTHF_250_-*b*-PCL_10_	PTHF_250_	16	10	11.4	1390	1560	18	61	33,41	63	46
PCL-*b*-PTHF_250_-*b*-PCL_15_	PTHF_250_	12	15	16.2	1960	2100	18	70	39,41	74	54
PCL-*b*-PTHF_250_-*b*-PCL_20_	PTHF_250_	10	20	20.3	2530	2570	22	65	41,47	72	53
PPG_425_		—	—	6.8	425	396			−71^l^	—	—
PCL-*b*-PPG_425_-*b*-PCL_5_	PPG_425_	43	5	7.8	1000	1280	−15	37	2,20	33	24
PCL-*b*-PPG_425_-*b*-PCL_10_	PPG_425_	27	10	14.2	1570	2020	11	57	28,39	56	41
PCL-*b*-PPG_425_-*b*-PCL_15_	PPG_425_	20	15	18.7	2130	2530	18	62	38,45	62	45
PCL-*b*-PPG_425_-*b*-PCL_20_	PPG_425_	16	20	22.7	2680	2980	20	60	40,48	61	45

a Obtained from % ether = (MW_initiator_/*M*_n_(NMR)) × 100; where MW_initiator_ is the molecular weight of initiator (HOPEGOH, HOPTHFOH, or HOPPGOH).

b Determined by ^1^H NMR in CDCl_3_

c Obtained from CL/polyether feed molar ratio.

d Using end-group analysis by ^1^H NMR.

e Obtained from the equation *M*_n_(calcd) = (MW(CL))·(mmol CL/mmol polyether) + MW(polyether), where MW is the molecular weight of ε-caprolactone (CL, 114 g mol^−1^) monomer or initiator.

f Obtained from *M*_n_(NMR) = (DP(NMR) × MW(repetitive unit)) + MW(polyether).

g Obtained by DSC analysis (first cooling).

h Obtained by DSC analysis (second heating).

i Using the value of 135.3 J g^−1^ for a PCL 100% crystalline,^68^ the crystallinity of PCL (*x*_PCL_) was calculated.

j Reported value for PEG (*M*_n_ = 200 g mol^−1^), liquid at room temperature.^70^

k *T*_m_ of the PTHF segment.

l Reported value for PPG (*M*_n_ = 425 g mol^−1^), liquid at room temperature.^71^

m Enthalpy of fusion attributed to the PCL, obtained by the equation Δ*H*_mPCL_ = (Δ*H*_m_) × (weight fraction of PCL).

Trifluoroacetic anhydride (TFAA) was used to derivatize all samples to visualize the repeating unit and end groups in the triblock copolymers. This was done to prevent overlapping peaks, as in the case of PCL-*b*-PEG-*b*-PCL triblock copolymers derived from PEG and PCL regarding the signals between the methylene attached to the hydroxyl [–CH_2_OH] and methylene from the ether groups [–OCH_2_CH_2_OCH_2_CH_2_O–]. Ultimately, trifluoroacetate ester groups [–CH_2_O(C=O)CF_3_] were produced.^66^

**Fig. 1:**
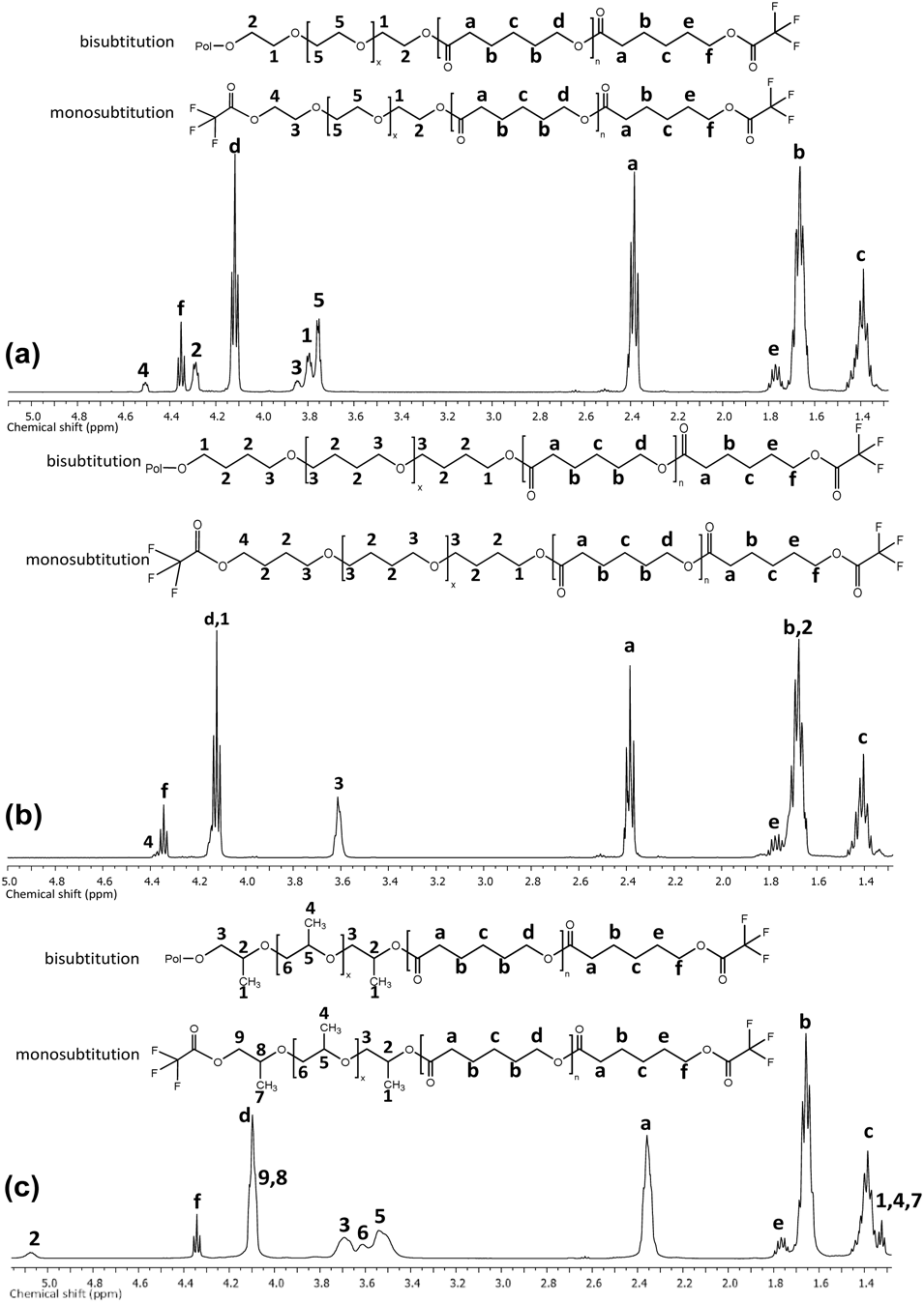
^1^H (400 MHz) spectra in CDCl_3_ at room temperature for copolymers (a) PCL-*b*-PEG_200_-*b*-PCL_10_ (*M*_n_ (NMR) = 1 530, DP = 10), (b) PCL-*b*-PTHF_250_-*b*-PCL_10_ (*M*_n_ (NMR) = 1 560, DP = 10), and (c) PCL-*b*-PPG_425_-*b*-PCL_10_ (*M*_n_ (NMR) = 2020, DP = 10).

**Fig. 2:**
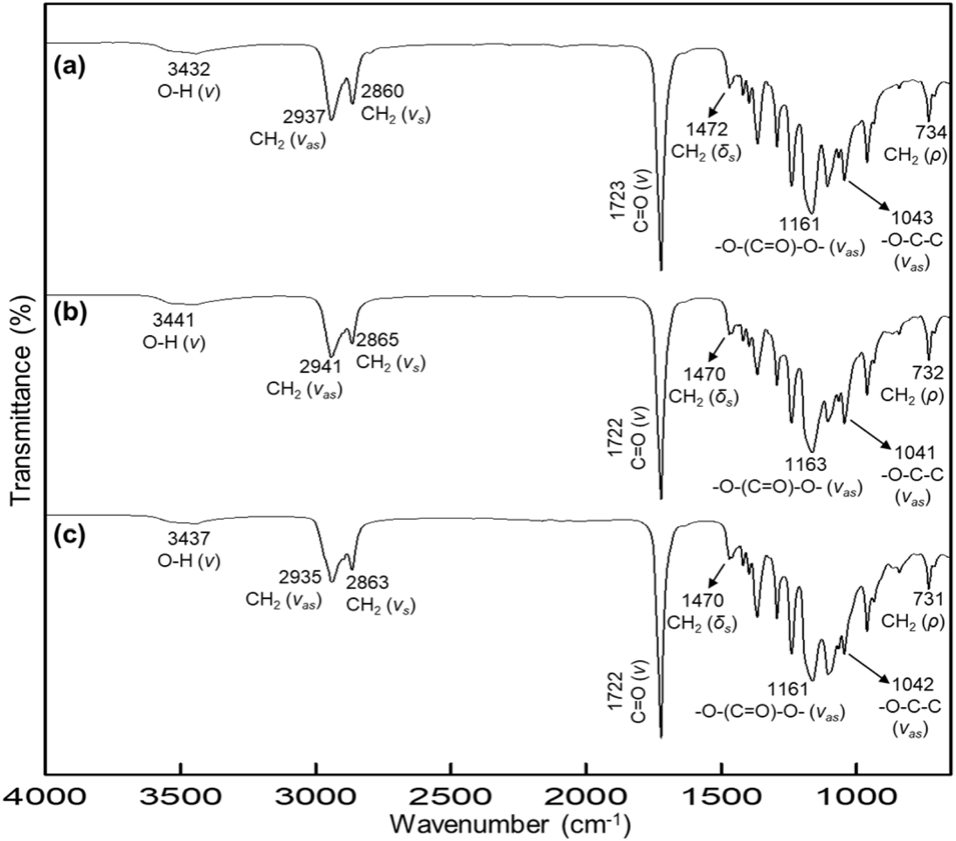
FTIR spectra for triblock copolymers with DP = 10: (a) PCL-*b*-PEG_200_G-*b*-PCL_10_, (b) PCL-*b*-PTHF_250_-*b*-PCL_10_, and (c) PCL-*b*-PPG_425_-*b*-PCL_10_.

Fig. 1 shows the ^1^H NMR spectra for three different samples of triblock copolymers where segment B (macroinitiators) was changed in the PCL main chain. The PCL-*b*-PEG-*b*-PCL signals (Fig. 1a) of the methylenes derived from polyethylene glycol as a macroinitiator were now appear as part of the PCL backbone in two modes: (a) bisubstitution [PCL–O–CH_2_(2)–CH_2_(1)–O–[CH_2_(5)–CH_2_(5)–O]_*x*_–CH_2_(1)–CH_2_(2)–O–PCL] and (b) monosubstitution [H–O–CH_2_(4)–CH_2_(3)–O–[CH_2_(5)–CH_2_(5)–O]_*x*_–CH_2_(1)–CH_2_(2)–O-PCL]. The FT-IR spectra for triblock copolymers show six characteristics bands at 3441–3432 (*ν*, –OH), 2941–2935 (*ν*_as_, –CH_2_−), 2865–2860 (*ν*_s_, –CH_2_−), 1722 (*ν*, C=O), 1161 (*ν*_as_, –C–(C=O)–O–), and 734–731 (*ρ*, –CH_2_−) cm^−1^ (Fig. 2).

**Fig. 3:**
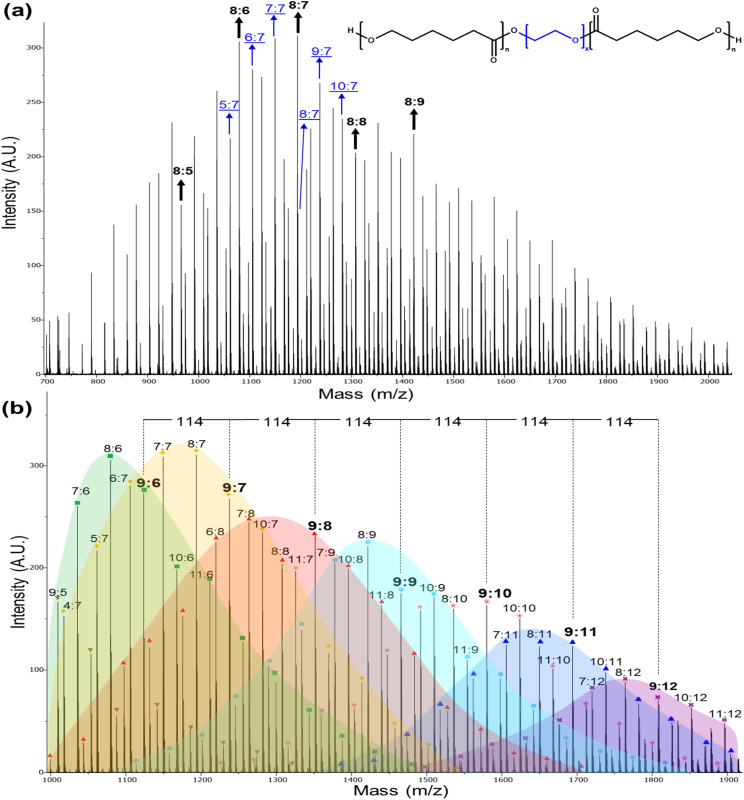
MALDI-TOF spectra (reflector mode) for PCL-*b*-PEG_1000_-*b*-PCL_10_ triblock copolymer. Where the numbers (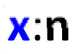) correspond to the DPs of different species of ethylene glycol (–CH_2_–CH_2_–O–)_*x*_ and ε-caprolactone (CL) [–CO(CH_2_)_5_O–]_*n*_ units in the triblock copolymer, respectively (note: 114 and 44 are the values of the molecular weights of CL and –CH_2_–CH_2_–O–, respectively). (a) Complete view from 700–2050 *m*/*z* fragments. (b) Expanded view from 995–1920 *m*/*z* fragments doped with Na^+^. DP_PCL_ = 5 (inverted triangle), 6 (square), 7 (rhombus), 8 (triangle), 9 (circle), 10 (star), 11 (trapezoid), and 12 (cross). Note: Distribution with DP = 10 (star) was not colored to facilitate the observation of curves.

MALDI-TOF spectrometric analysis is a powerful analytical technique to visualize repeating units, molecular weight distributions, and terminal groups. The *m*/*z* peaks that were measured in the MALDI-TOF spectra were then compared to the theoretical masses of the repeating units [CL or macrodiol (PEG, PTHF or PPG)] and cations attached to the polymeric chain (Na^+^ or K^+^). A larger DP of PCL resulted in representative spectra that exhibit increased complexity. Thereby facilitating the reproducible assignment of polymer chains and clear visualization of polymer dispersion. The MALDI-TOF mass spectrum of triblock polymer PCL-*b*-PEG_1000_-*b*-PCL_10_ [*M*_n(NMR)_ = 2000 g mol^−1^] showed different distributions separated by 114 and 44 mass units, which corresponded to the molecular weights of the CL and –CH_2_CH_2_O– comonomers, respectively (Fig. 3a). DP_PCL_ for a few species is indicated by bold numbers (ethylene glycol in PEG (EG)/PCL = 8: 5–9) at the top of the MALDI-TOF curve, and DP_PEG_ is indicated by blue underlined numbers (ethylene glycol in PEG/PCL = 5–10: 7).

An expanded view of the MALDI-TOF mass spectrum for PCL-*b*-PEG_1000_-*b*-PCL_10_ fragments (Fig. 3b) showed repeating units between 1000 and 1900 Da. The distribution of each DP of the PCL block (DP_PCL_ = 6–12) is shown in a different color in the figure and exhibits variations in the number of ethylene glycol in PEG in the block (DP_PEG_ = 1–16). For example, fragments marked with a blue circle match DP_PCL_ = 9 (this number of PCL units in the polymer chain being constant), beginning with the first circle for fragment 1113 (EG/PCL = 1 : 9), this represented the addition of 1 unit of ethylene glycol to the polymer chain. Then, followed by 1157 (EG/PCL = 2 : 9), 1201 (EG/PCL = 3 : 9), and so on, forming the corresponding distribution until 1818 (EG/PCL = 17 : 9), which is marked with light-blue shading (Fig. 3b).

The distribution designated by yellow shading and rhombus corresponded to DP_PCL_ = 7 (with a constant of 7 PCL units in the polymer chain). The initial fragment of 1018 was assigned to a triblock containing 4 units of ethylene glycol in the polymer chain attached to PCL (EG/PCL = 4 : 7). The next fragment of 1061 is the addition of 1 more unit of ethylene glycol (EG/PCL = 5 : 7). The process was repeated, thereby establishing the corresponding distribution to the smallest fragments of 1543 (EG/PCL = 16 : 7), thus completing the yellow coloration (Fig. 3b).

### 3.2 Part 2. effect of segment A (PCL)

**Fig. 4:**
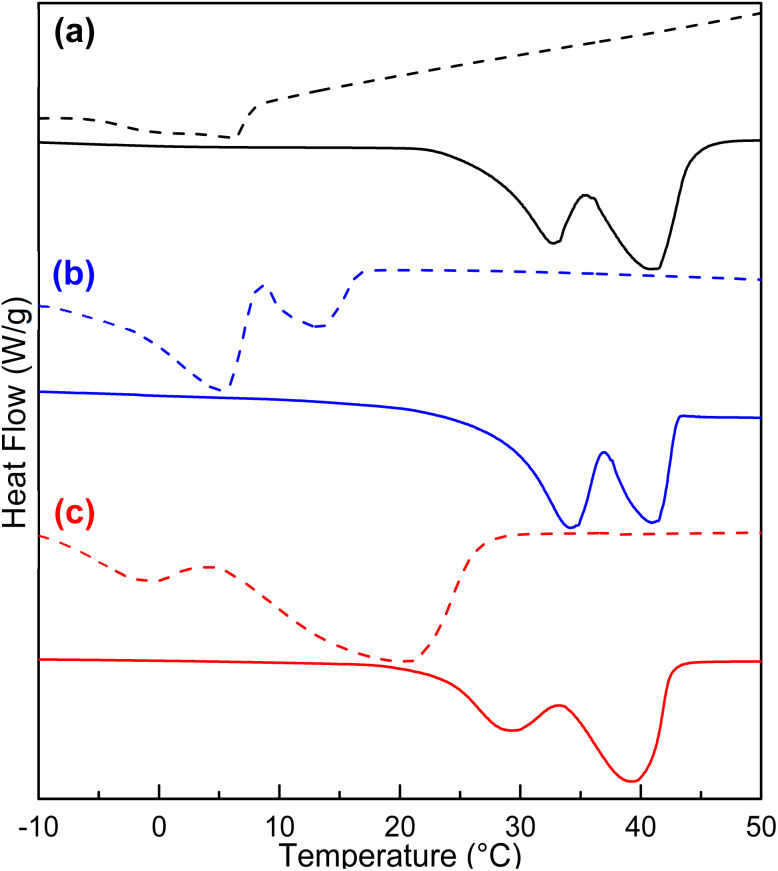
DSC thermograms (a) PCL-*b*-PEG_200_-*b*-PCL, (b) PCL-*b*-PTHF_250_-*b*-PCL, and (c) PCL-*b*-PPG_425_-*b*-PCL. Dashed line corresponds to DP = 5; solid line corresponds to DP = 10.

DP of the poly(ε-caprolactone) affected the triblock copolymers by increasing the crystallinity values of the PCL segment (Table 3). In the case of the triblock copolymer PCL-*b*-PPG_425_-*b*-PCL, DSC thermograms exhibited a regular pattern for all samples. The double endothermic transition represents the crystalline domain of the PCL segment involving two different sizes of crystallites. Only *T*_m_ of the PCL segment (between 20 and 48 °C) was observed for DP = 5, 10, 15, and 20. Fig. 4 shows DSC thermograms to compare the triblock copolymers species PCL-*b*-PEG_200_-*b*-PCL, PCL-*b*-PTHF_250_-*b*-PCL, and PCL-*b*-PPG_425_-*b*-PCL. These thermograms exhibited two endothermic peaks for the PCL, which indicated the presence of two distinct crystallite sizes originating from two distinct environments. These environments could be crystallites found in amorphous domains and the remainder found in more crystalline environments exhibiting relatively higher *T*_m_ (41 °C), as shown by the solid line for PCL-*b*-PEG_200_-*b*-PCL_10_ in Fig. 4a.

**Fig. 5:**
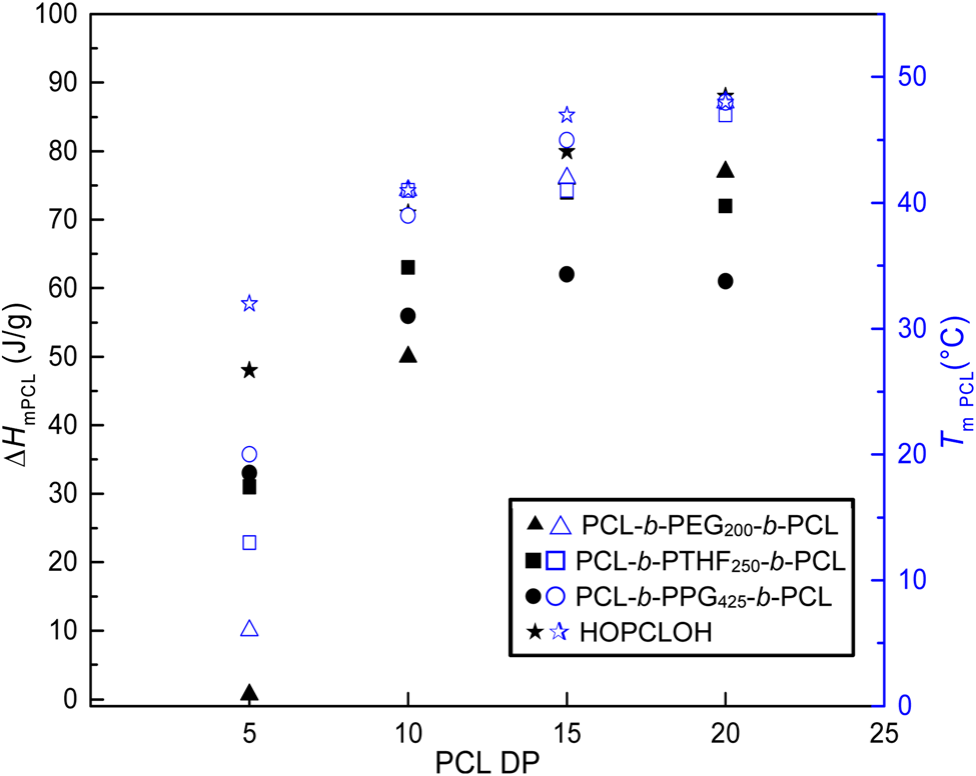
Effect of PCL block length on enthalpy (Δ*H*_m_) and temperature (*T*_m_) of ABA triblock copolymers PCL-*b*-PEG_200_-*b*-PCL, PCL-*b*-PTHF_250_-*b*-PCL, PCL-*b*-PPG_425_-*b*-PCL and for homopolymer HOPCLOH. For enthalpy, filled figures (Δ*H*_m_: ▴■●★) and for *T*_m_, blue open figures (*T*_m_: 
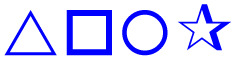
).

*T*_m_ increased proportionally with DP. For PCL-*b*-PTHF_250_-*b*-PCL, the values of the two endothermic peaks increased by 5 and 13 °C (DP = 5), 33 and 41 °C (DP = 10), 39 and 41 °C (DP = 15), and 41 and 47 °C (DP = 20). The remaining triblock copolymers in Table 2 exhibited a comparable effect (PCL-*b*-PEG_200_-*b*-PCL and PCL-*b*-PPG_425_-*b*-PCL), as did all the families of triblock copolymers (Tables S4, S5, and S6). Regardless of the type of macroinitiator, both *T*_m_ and the enthalpy of fusion (Δ*H*_m_) increased with the DP of PCL, as shown in Fig. 5. The profile of crystallization exhibited a comparable value of enthalpy of crystallization (Δ*H*_c_) with respect to the enthalpy of fusion (Δ*H*_m_), suggesting a reversible process (Table 3 and Fig. S4). The first heating was comparable with respect to the second heating, suggesting that the thermal history did not significantly influence the observed results (Fig. S4).

The *T*_m_ and Δ*H*_m_ values of the triblock copolymers were compared with those of a homopolymer of PCL and 1,8-octanediol (HOPCLOH) (Fig. 5, star). It was evident that HOPCLOH had higher Δ*H*_m_ and *T*_m_ than copolymers with identical DP. This observation was attributable to the reduced interaction of the PCL fraction contained within the triblock copolymers with the B block, which resulted from the increased length of the main chain. This led to the formation of an independent semicrystalline PCL domain. Consequently, the differences decreased as the DP of PCL increased.

The values of the triblock copolymers were compared with those of other PCL-diols of similar molecular weights reported in previous publications (between 600 and 3000 g mol^−1^) as shown in Table 4. *T*_m_ and Δ*H*_m_ values for the lower *M*_n_ were 17 °C and 66 J g^−1^.^72^ For the higher *M*_n_, the *T*_m_ and Δ*H*_m_ values were 57 °C and 88 J g^−1^, respectively.^72–75^ Comparing the three systems PCL-B-PCL, where B = PEG, PTHF, or PPG, and similar species previously published (Table S7) we can observe similar behavior in terms of thermal properties.

**Table 4:** Comparison with PCL-diols with similar molecular weights

Initiator	PCL-diol *M*_n_ (g mol^−1^)	*T*_m_ (°C)	Δ*H*_m_ (J g^−1^)	Author and year
HO(CH_2_)_*m*_OH, where *m* = 4,8,12	600	17	66	Barrera-Nava, 2024 ref. 72
	1300	34	81	
	1800	41	88	
None	2000	57	75	Erceg, 2023 ref. 73
Ethylene glycol	1000	31,36	—	Choi, 2023 ref. 74
	2400	46,48	—	
	3000	45,49	—	
HO(CH_2_)_*m*_OH, where *m* = 2–16	900	38	85	Báez, 2017 ref. 75
	1150	44	100	

**Fig. 6:**
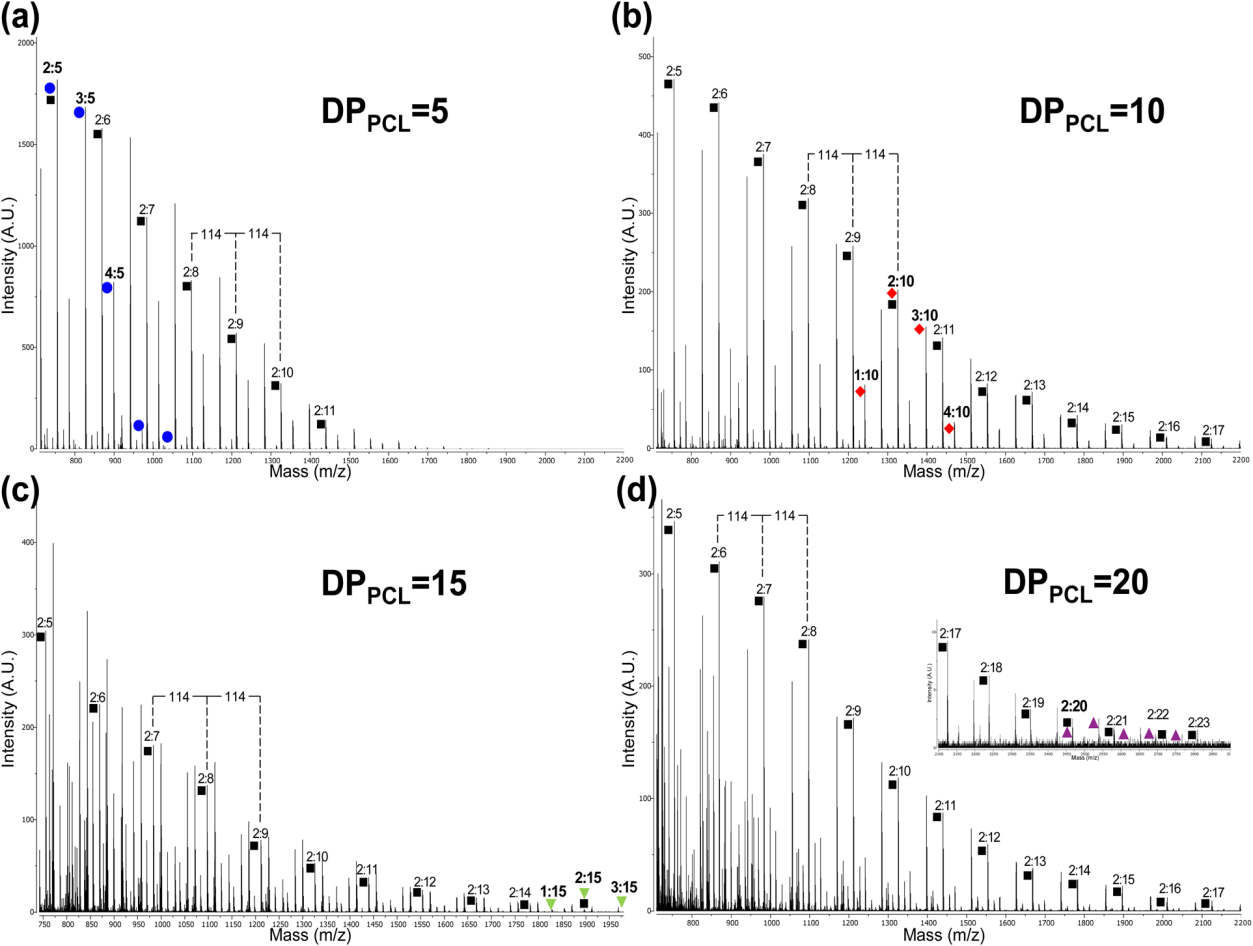
MALDI-TOF spectra (reflector mode) of PCL-*b*-PTHF_250_-*b*-PCL triblock copolymers with different degrees of polymerization of the PCL block (DP_PCL_ = 5, 10, 15, and 20), obtained in the range of 710 to 2200 *m*/*z* for (a and b); range of 740 to 1975 *m*/*z* for (c); and 700 to 3000 *m*/*z* for (d). Numbers (*x* : *n*) correspond to the DPs of different species of tetramethylene oxide (–CH_2_–CH_2_–CH_2_–CH_2_–O–)_*x*_ and ε-caprolactone (CL) [–CO(CH_2_)_5_O–]_*n*_ units in the triblock copolymer, respectively (note: 114 and 72 are the values of the molecular weights of CL and –(CH_2_)_4_–O–, respectively). Colored figures correspond to the indicated DP_PCL_.

The MALDI-TOF analysis results of PCL-*b*-PTHF_250_-*b*-PCL triblock copolymers with different DP are presented in Fig. 6. As the PCL DP increased, both the signal density and complexity also increased. Greater diversity of oligomeric species was present when DP was higher, as evidenced by the presence of more peaks within the same range. This was consistent with a broader polymer distribution characteristic of systems with more repeating units.

Furthermore, the normal separation of the caprolactone unit (114 g mol^−1^) was maintained, and the increment in molecular mass was confirmed. Most of the strong peaks were concentrated at the beginning of the range at DP = 5 (Fig. 6a). As the DP increased (DP = 10, 15, and 20), the peaks exhibited a shift toward higher *m*/*z* closer to 2000–2200 (Fig. 6b–d).

### 3.3 Part 3. segment B (PEG, PTHF or PPG)

#### 3.3.1 Effect of type of segment B

**Fig. 7:**
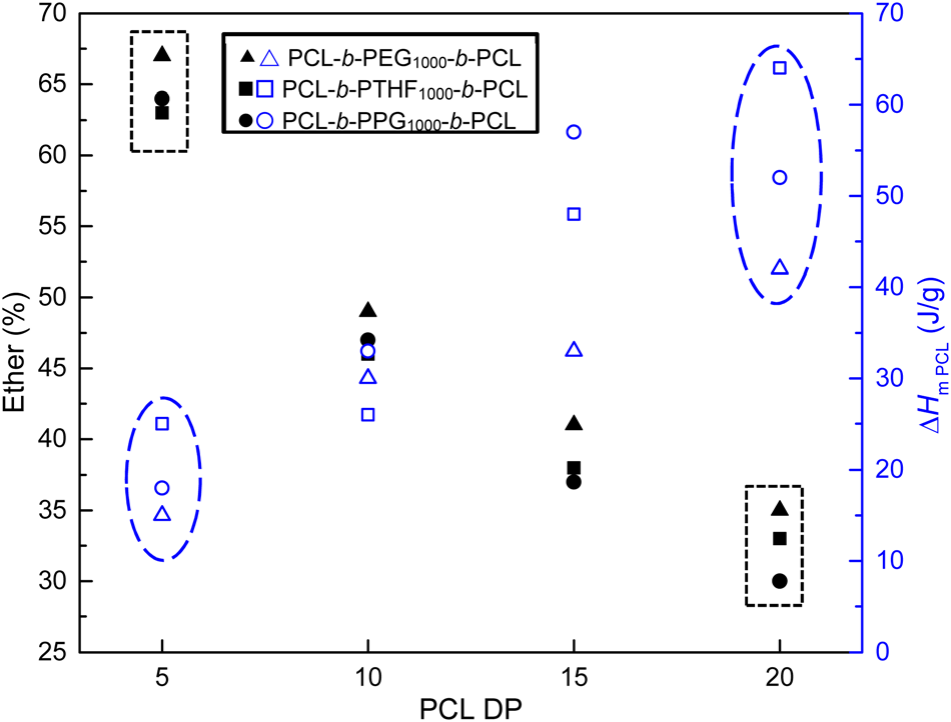
Relation of PCL block length with ether content (%) and enthalpy (Δ*H*_m_) of ABA triblock copolymers PCL-*b*-PEG_1000_-*b*-PCL, PCL-*b*-PTHF_1000_-*b*-PCL, and PCL-*b*-PPG_1000_-*b*-PCL. For ether content, filled figures (ether (%): ▴■●) and for Δ*H*_mPCL_, blue open figures (Δ*H*_mPCL_: 
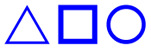
). Note: the dashed blue oval and black rectangle illustrate an inversely proportional relationship between the ether percentage and Δ*H*_m_.

Usually, the selection of segment B for triblock or diblock copolymers involves consideration of a series of significant effects on the physical properties, such as crystallinity,^76,77^ thermal stability,^78–80^ self-assemby,^81,82^ lithography,^83^ supramolecular interactions,^84–86^*etc.*Fig. 7 shows the dramatic differences between the types of polyether block segment. The enthalpy of fusion of the semicrystalline domains of PCL values decreased for DP_PCL_ = 5 in the following order with respect to the B segment: PPG < PEG < PTHF. The family of triblock copolymers derivatives from PCL-*b*-PPG-*b*-PCL_10_ had the most remarkable gap of Δ*H*_m_ between DP_PCL_ = 5 and 20, which can be attributed to the incompatibility and immiscibility of both segments favoriting better segregation of phases. This effect was attributed to: (1) the steric hindrance of the methyl branch of the PPG and (2) the flexibility of the ether group with regard to the number of atoms per repeating unit: –(CH_2_)_4_–O–[PTHF] *versus* –(CH_2_)_2_–O–[PEG].

Nevertheless, all polyether B segments (PEG, PTHF, and PPG) acted as plasticizers and disrupted the semicrystalline microdomains of segment A. This decreased the enthalpy of fusion (Δ*H*_mPCL_) in an inversely proportional manner, as illustrated in Fig. 7 (ovals and rectangles). In most cases, the disruption of the crystallinity was greater with PEG block according to the Δ*H*_m_ results. When PTHF was used as the B segment, it generated less disorder within the crystalline microdomain of PCL environment of the A block. PTHF as the B segment gradually exhibited a predisposition to yield optimal crystallinity without compromising flexibility and improve phase segregation, which could be observed as a signal in the DSC thermograms (which will be discussed in next section). Using PPG as segment B significantly reduced the crystallinity of the PCL block (Tables 3 and S4–S6) in comparison with PEG or PTHF as the B segment, and it acted as a better plasticizer for PCL.

**Fig. 8:**
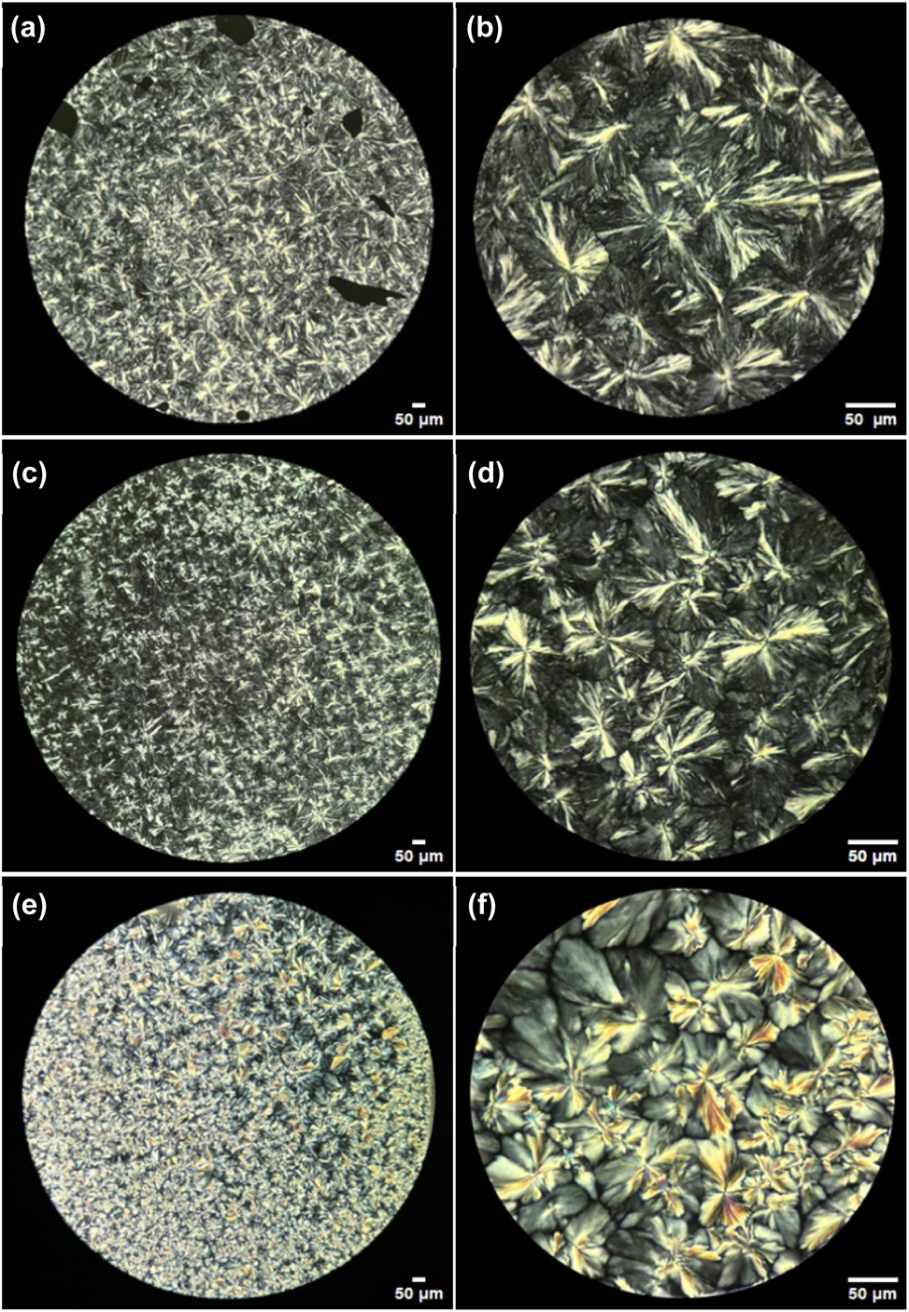
Polarized optical microscopy (POM, magnification: 10× (left) and 40× (right)) of (a and b) PCL-*b*-PEG_200_-*b*-PCL_10_, (c and d) PCL-*b*-PTHF_250_-*b*-PCL_10_, and (e and f) PCL-*b*-PPG_425_-*b*-PCL_10_.

To visualize the physical form of the triblock copolymers, Fig. 8 presents the polarized optical microscopy (POM) results. For PCL-*b*-PEG_200_-*b*-PCL_10_, spherulites with a well-defined radial structure were observed (Fig. 8a and b). Similar spherulites were detected for PCL-*b*-PTHF_250_-*b*-PCL_10_, which had a higher density of smaller spherulites (Fig. 8c and d). This suggested that using PHTF as segment B promoted nucleation but limited spherulitic growth.

In the case of PCL-*b*-PPG_425_-*b*-PCL_10_, multiple spherulites with less defined morphology and characteristic color were observed (Fig. 8e and f). The change in color can be attributed to the methyl groups present in PPG. These methyl groups have been shown to increase chain irregularity, disrupt the lamellar packing of PCL segments, induce anisotropy, and produce a bathochromic shift in interference colors. Analogous effects have been reported for semicrystalline polyesters that have been plasticized with PPG and copolyesters that contain bulky or branched linkers. In these cases, the crystallization of the polyester has been reduced, and enhanced color has been observed under cross-polarized light.^87,88^

#### 3.3.2 Effect of length of segment

To compare the effect of the length of segment B in the triblock copolymers, three molecular weights of each type of macroinitiator (polyether) were used, considering the similar molecular weights between the types of macroinitiators employed. The molar mass of segment B directly influenced the melting temperature (*T*_m_), enthalpy of fusion (Δ*H*_m_), and crystalline content (*x*_*i*_) of the copolymers, as illustrated in Tables S4–S6. A clear trend was observed as the size of segment B increased, with *T*_m_ decreasing in the majority of cases (Fig. S5–S7).

**Fig. 9:**
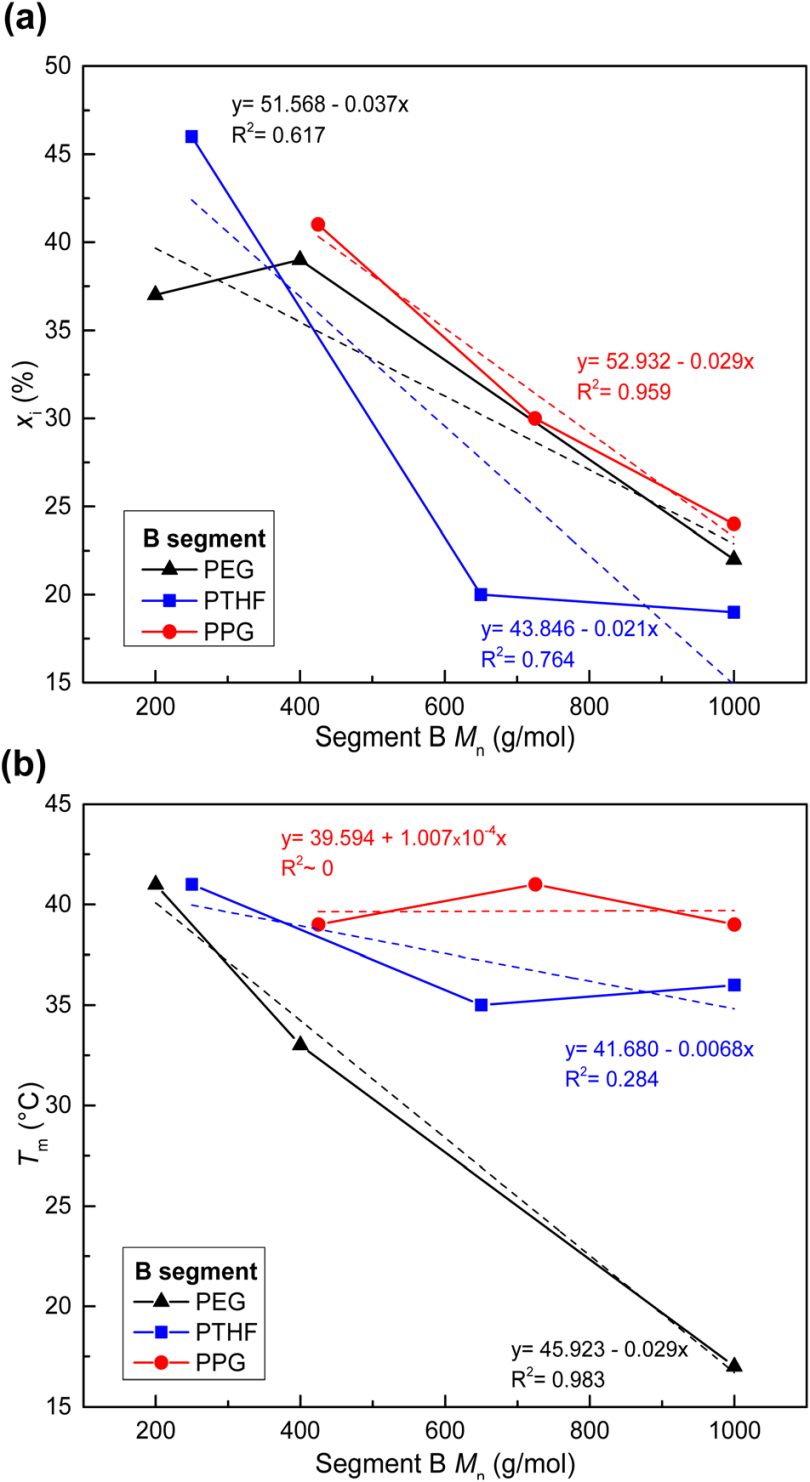
Effect of length of segment B in ABA triblock copolymers (a) crystallinity (*x*_i_) and (b) melting temperature (*T*_m_). Segment B: PEG (200, 400, 1000 g mol^−1^), PTHF (250, 650, 1000 g mol^−1^) y PPG (425, 725, 1000 g mol^−1^). DP_PCL_ = 10.

Copolymer PCL-*b*-PEG-*b*-PCL_10_ showed a decrease in *T*_m_ and *x*_*i*_ of the PCL segments as *M*_n_ of PEG increased from 200 to 400 and 1000 g mol^−1^ (Fig. 9a and b, triangles). This indicated enhanced chain flexibility and potential interference of the PEG with the crystalline arrangement of PCL at longer lengths. In the case of copolymers PCL-*b*-PTHF-*b*-PCL_10_ (Fig. 9a, squares) and PCL-*b*- PPG-*b*-PCL_10_ (Fig. 9a, circles), *x*_*i*_ exhibited a similar tendency with a more pronounced decrease in crystallinity compared to the copolymers with PEG as segment B.

The melting temperatures (*T*_m_) with PTHF as the central segment showed a slight decrease (Fig. 9b, squares), and those with PPG displayed minor fluctuations but maintained similar values (Fig. 9b, circles). In contrast, *T*_m_ of PCL-*b*-PEG-*b*-PCL showed a decrease when *M*_n_ of the PEG segment was higher (1000 g mol^−1^) in comparison with the rest of the samples. This was probably attributable to the major dispersity (*D*_M_) of the PEG macroinitiator producing triblock copolymers with heterogeneity of the B segment. The linear trend analysis demonstrated the interference of PEG as B segment, which exhibited a strong negative correlation (*R*^2^ = 0.983), suggesting a pronounced disruption of PCL semicrystalline microdomain. THF and PPG exhibited negligible or weak dependency (*R*^2^ = 0.284, and *R*^2^ ∼ 0, respectively).

**Fig. 10:**
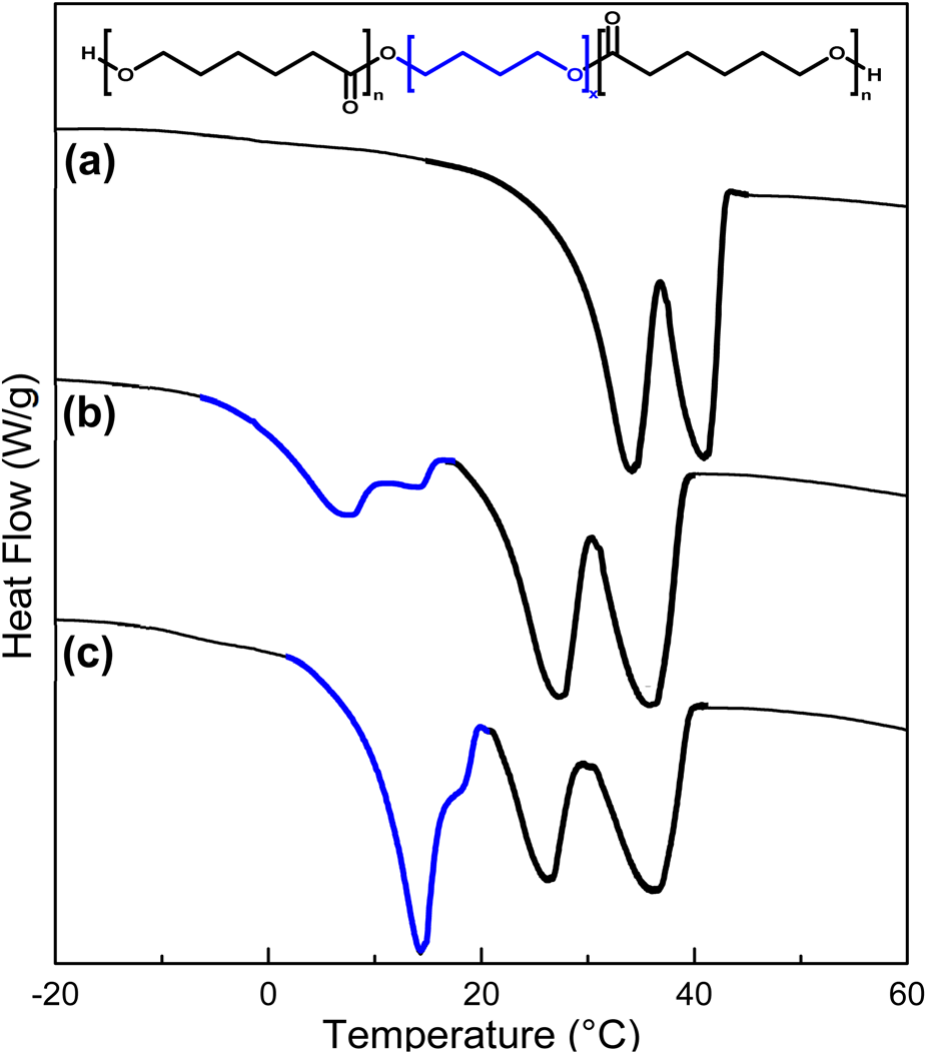
DSC thermograms (a) PCL-*b*-PTHF_250_-*b*-PCL, (b) PCL-*b*-PTHF_650_-*b*-PCL, and (c) PCL-*b*-PTHF_1000_-*b*-PCL. DP_PCL_ = 10.

Fig. 10 shows DSC thermograms to provide a better perspective of the thermal properties for PCL-*b*-PTHF-*b*-PCL copolymers. A proportional increment of the signal was distinctly evident in the zone belonging to the segment B. At the lowest molecular weight of the PTHF segment (250 g mol^−1^), the curve of the PTHF fusion signal was not visible due to its amorphous nature and liquid state at room temperature (25 °C) (Fig. 10a). As the weight of the PTHF segment increased to 650 g mol^−1^ (Fig. 10b, blue segment), a signal was observed at 7 °C. Finally, with higher molecular weight of the segment (1000 g mol^−1^), the *T*_m_ signal was observed at 14 °C with more intensity (Fig. 10c, blue segment), this finding was consistent with other reports on copolymers with PTHF and PCL.^53^ Thus, PCL-*b*-PTHF-*b*-PCL showed segregation of phases of two microcrystalline domains according to the DSC analysis, which was consistent with the semicrystalline domains shown by POM in Fig. 8c and d.

The presence or absence of a *T*_m_ signal of PTHF segment in the DSC thermograms was attributed to the balance between the PTHF molecular weight and the nature/length of the terminal A blocks. The PTHF block has been observed to form independent crystalline domains and produce a detectable *T*_m_ when it possesses sufficiently high *M*_n_. However, when the A segments exhibited high hydrophilicity (*e.g.*, PEG or PMeOX) and were of comparable length with the PTHF, they restricted the mobility and domain formation of the central PTHF block, thereby suppressing its crystallization.^89^ Conversely, when A segment was hydrophobic (*e.g.*, PLA or in this case, PCL), the thermal behavior can be dominated by the A-block, thereby concealing PTHF melting.^90,91^ This effect was only overcome when the PTHF domain size became large enough to overcome the confinement imposed by the ABA architecture.

**Fig. 11:**
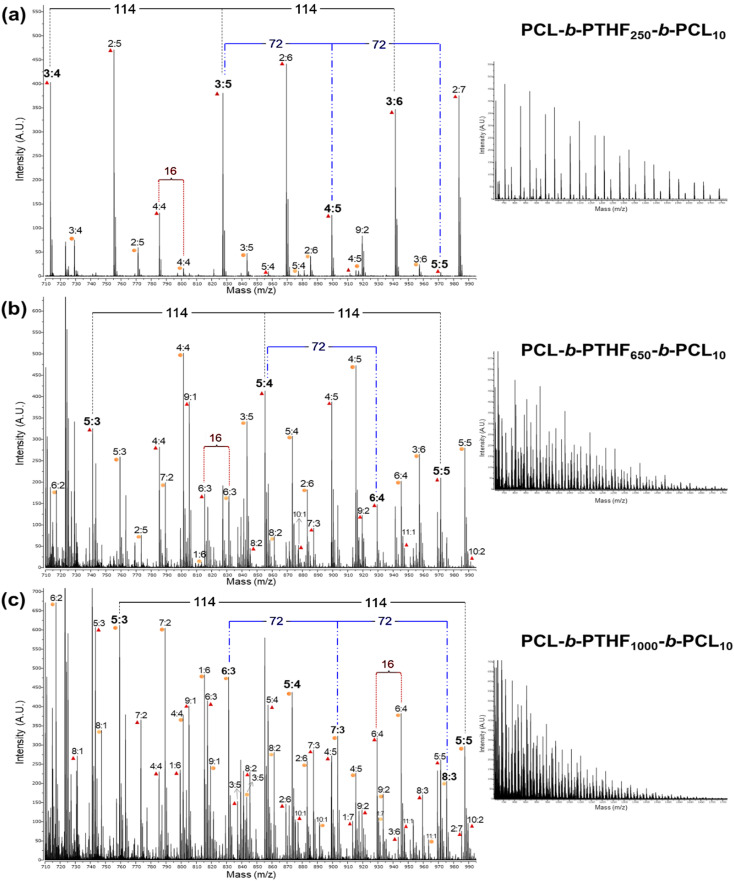
MALDI-TOF spectra (reflector mode) of PCL-*b*-PTHF-*b*-PCL_10_ triblock copolymers with different lengths of segment B. Where the numbers (*x* : *n*) correspond to the DPs of different species of tetramethylene oxide (–CH_2_–CH_2_–CH_2_–CH_2_–O–)_*x*_ and ε-caprolactone (CL) [–CO(CH_2_)_5_O–]_*n*_ units in the triblock copolymer, respectively (note: 114 and 72 are the values of the molecular weights of CL and –(CH_2_)_4_–O–, respectively). (a) PCL-*b*-PTHF_250_-*b*-PCL_10_, (b) PCL-*b*-PTHF_650_-*b*-PCL_10_ and (c) PCL-*b*-PTHF_1000_-*b*-PCL_10_. On the left, expanded view from 710–995 *m*/*z*, on the right, complete view from 710–1775 *m*/*z*. Fragments doped with K^+^ are indicated with an orange circle and fragments doped with Na^+^ are indicated with a red triangle.

The MALDI-TOF spectrum also provided evidence of the chemical nature in terms of both blocks (ABA) and the effect of increasing the length of block B. Fig. 11 shows the MALDI-TOF spectra of the copolymers PCL-*b*-PTHF_250_-*b*-PCL_10_, PCL-*b*-PTHF_650_-*b*-PCL_10_, and PCL-*b*-PTHF_1000_-*b-*PCL_10_. In each case, an expanded view of the spectrum is presented on the left to show the details of the signal separation. The complete spectrum is presented on the right to show the overall effect of the length of B segment. In all spectra, signals spaced 114 and 72 Da apart were observed, which corresponded to repeating CL and PTHF units, respectively.

The signals that were 16 Da apart corresponded to the difference between complexes formed with Na^+^ or K^+^. When increasing the length of the B segment, the spectrum became more complex, showing higher signal density and a dispersed pattern (Fig. 11c) compared to the spectrum obtained with lower length of the segment B (Fig. 11a). The increase in the number of signals can be attributed to the more varied combinations between blocks and a wider distribution of architecture.

### 3.4 Part 4. poly(ester urethane)s (PEUs) derived from ABA triblock copolymers

**Scheme 2:**
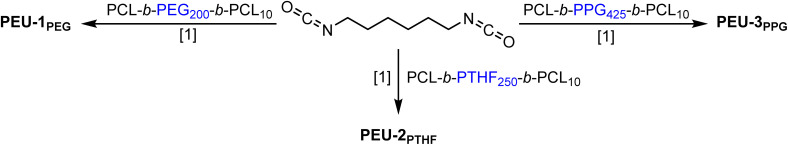
Synthesis of poly(ester urethane)s (PEUs) derived from ABA triblock copolymers with DP = 10. Note [1] = 80 °C, 3 hours, 3 drops of Sn(Oct)_2_ and 10 mL of 1,2-dichloroethane (DCE).

Three different PEUs were synthesized using the triblock copolymers and 1,6-hexamethylene diisocyanate (HDI) (Scheme 2) and analyzed by FTIR spectrometry. The spectra for PEU-3_PPG_ (Fig. S10) presented bands at 3347, 1620, and 1533 cm^−1^, which are characteristic of urethane group vibrations (–O–C=O–NH) with N–H (*ν*, stretching), C=O (*ν*, stretching), and N–H (*δ*, bending), respectively. A band was identified for the carbonyl of the ester group in PCL (1722 cm^−1^, *ν*, stretching). In the three PEU samples, carbonyl group signals of unreacted HDI (∼2250 cm^−1^) were not detected, which confirms the formation of urethane groups (Fig. S8–S10). The ^1^H NMR spectrum confirmed the presence of PEU-3_PPG_ (Fig. S11) and exhibited signals at 3.13 and 1.47 ppm, which were assigned to methylenes of the urethane chain [HN–CH_2(*x*)_–CH_2(*y*)_–].

Using DSC analysis, the effects of the three different types of segment B were examined through their thermal properties and physical properties. The melting temperature (*T*_m_) of all the PEUs was affected by the semicrystalline domain of PCL segments, and the three PEUs showed similar values between 30 and 33 °C (Table 5). Using PPG as the B block led to the lowest crystallinity (*x*_i_ = 20%). This was consistent with the results of the triblock copolymer precursor, where PPG generated the most disruptions in the semicrystalline microdomains of PCL segments.

**Table 5:** Thermal and mechanical properties of poly(ester urethane)s (PEUs) prepared from triblock copolymers (PCL-*b*-B-*b*-PCL) and hexamethylene diisocyanate (HDI)

Polyurethane	Precursor	Ether (%)^a^^,^^b^	HS (%)^c^	SS (%)^d^	*T*_mPCL_ (°C)^e^	Δ*H*_m_ (J g^−1^)^e^	*x*_*i*_ (%)^e^^,^^g^	Stress at break (MPa)	Strain at break (%)	Modulus (MPa)
PEU-1_PEG_	PCL-*b*-PEG_200_-*b*-PCL_10_	14	13	87	31	38 (50)^f^	28 (37)^f^	2.7 ± 0.2	3 ± 0.3	133.5 ± 2.0
PEU-2_PTHF_	PCL-*b*-PTHF_250_-*b*-PCL_10_	16	13	87	33	30 (63)^f^	22 (46)^f^	3.3 ± 0.1	14 ± 0.7	78.4 ± 2.9
PEU-3_PPG_	PCL-*b*-PPG_425_-*b*-PCL_10_	27	12	88	30	28 (56)^f^	20 (41)^f^	2.7 ± 0.1	8 ± 0.8	85.6 ± 1.2

a Obtained from the equation % ether = (MW_initiator_/*M*_n_(NMR)) × 100; where MW_initiator_ is the molecular weight of initiator (HOPEGOH, HOPTHFOH, or HOPPGOH).

b Determined by ^1^H NMR in CDCl_3_.

c Hard Segment.

d Soft Segment.

e Obtained by DSC analysis (second heating).

f The values enthalpy (Δ*H*_m_) and crystallinity (*x*_i_)of the triblock copolymer utilized for the PEU are indicated within parentheses.

g Using the value of 135.3 J g^−1^ for a PCL 100% crystalline,^68^ the crystallinity of PCL (*x*_PCL_) was calculated.

**Fig. 12:**
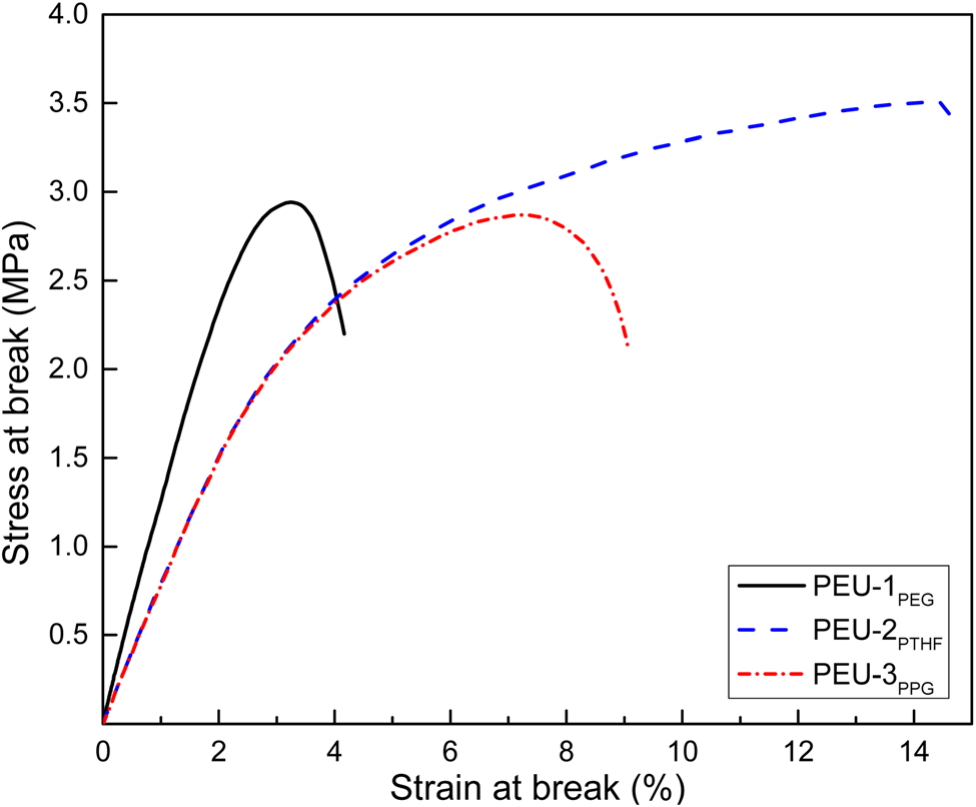
Stress–strain curves for PEUs derived ABA triblock copolymers with DP_PCL_ = 10 and 1,6-hexamethylene diisocyanate (HDI).

The mechanical properties of PEUs are exhibited on Fig. 12. The graphs of strain *versus* stress of the three different PEUs overlapped. A clear pattern of plastic behavior was observed where low values of stress at break and elongation at break indicated a fragile material. This was consistent with low modulus values and was attributed to the soft segment in the polyether block favoring an amorphous environment and less crystallinity (Table 5).

## 4 Conclusions

Different ABA triblock copolymers were prepared by ROP of ε-caprolactone (as block A) using three different macroinitiators as central segments. This study expands upon prior studies by emphasizing how the length and type of the center segment affect the thermal and morphological characteristics of ABA triblocks. The effects of the DP of block A, the type of macroinitiator, and their number-average molecular weights were examined. In all triblock copolymers, the crystallinity (*x*_i_) and melting temperature (*T*_m_) increased proportionally to the DP of PCL. As DP increased, the signal density, polymer distribution, and complexity increased in the MALDI-TOF spectra. Using PPG as segment B significantly reduced *x*_i_ of the PCL blocks compared to PEG or PTHF. As a result, PPG was considered a more effective plasticizer for PCL.

POM showed that PCL-*b*-PPG-*b*-PCL copolymers presented a color variation and less defined morphology in comparison to copolymers with PEG or PTHF, resulting in significant disruption in the crystalline microdomains of the PCL block. This was attributed to the branched methyl of the PPG segment. The B segment's length also directly impacted *x*_i_ of the PCL-*b*-PEG-*b*-PCL copolymers, and as the portion of the central segment increased, the crystallinity and *T*_m_ values decreased. For PCL-*b*-PTHF-*b*-PCL copolymers, the influence was lower. In the DSC thermograms, as the PTHF length increased, a *T*_m_ signal related to PTHF appeared and induced the segregation of phases. In the case of PCL-*b*-PPG-*b*-PCL copolymers, the *x*_i_ and *T*_m_ values were not significantly impacted and remained similar.

Three PEUs were derived from 1,6-hexamethylene diisocyanate and PCL-*b*-PEG-*b*-PCL, PCL-*b*-PTHF-*b*-PCL, and PCL-*b*-PPG-*b*-PCL. The products showed a decrease in crystallinity due to the hydrogen bonding, which disrupted the soft segment. The PEUs showed plastic behavior with poor mechanical properties that indicated fragility.

The evidence indicates that the ABA triblock architecture can be directly translated into polyurethane-based materials, signifying their potential for utilization in biomedical devices or soft matrix materials for controlled release systems, among other applications requiring flexible, biodegradable, or tunable mechanical properties. Additionally, the triblock copolymers potentially can be used as an additive in lubricant oils (castor oil). Due to the amphiphilic character of both segments in the ABA triblock copolymer, these species are precursors of micelles and vesicles. These structures require thermally stable materials with *T*_m_ between 44–60 °C or thermo-responsive materials with *T*_m_ between 37–42 °C.

## Author contributions

Miriam Paola Barrera-Nava: investigation, validation, formal analysis, writing – original draft. Gerardo González García: characterization, financial support. José Bonilla Cruz: characterization, financial support. Kenneth J. Shea: characterization, finacial support. José E. Báez: conceptualization, characterization, supervision, writing – original draft, writing – review, funding acquisition.

## Conflicts of interest

There are no conflicts to declare.

## Supplementary Material

RA-015-D5RA06419H-s001

## Data Availability

The data supporting this article have been included as part of the supplementary information (SI). Supplementary information is available. See DOI: https://doi.org/10.1039/d5ra06419h.
