# Supplementary material for: ABA triblock copolymers prepared with poly(ε-caprolactone) and PEG, PTHF, and PPG macroinitiators as the central segment: synthesis, characterization, and thermal properties
Source: RSC Adv. 2025 Nov 7;15(51):43405–20. doi: 10.1039/d5ra06419h (PMC12593421; doi:10.1039/d5ra06419h)
Supplement: RA-015-D5RA06419H-s001 [file RA-015-D5RA06419H-s001.pdf]

Supplementary Information (SI) for RSC Advances.
This journal is © The Royal Society of Chemistry 2025

Supplementary Information

ABA triblock copolymers prepared with poly(ε-caprolactone) and PEG, PTHF, and PPG macroinitiators as the central segment: synthesis, characterization, and thermal properties

Miriam P. Barrera-Nava, [a] Gerardo González García, [a] José Bonilla Cruz, [b] Kenneth J. Shea, [c] and José E. Báez *[a]

[a]Department of Chemistry, University of Guanajuato (UG), Noria Alta S/N, 36050 Guanajuato, Gto, Mexico.
[b]Advanced Functional Materials & Nanotechnology Group, Centro de Investigación en Materiales Avanzados S. C. (CIMAV-Unidad Monterrey), Av. Alianza Norte 202, Autopista Monterrey-Aeropuerto Km 10, PIIT, Apodaca-Nuevo León 66628 C.P., Mexico
[c]Department of Chemistry, University of California, Irvine (UCI), California 92697-2025, United States.

*Correspondence to: Prof. José E. Báez. Email: jebaez@ugto.mx

Table of contents

		Page
	Data characterization	4
**Tables**		
	**Table S1.** Triblock copolymers (PCL-*b*-PEG_x-*b*-PCL) prepared using polyethylene glycol (PEG) of different molecular weight [M_n= 200, 400, and 1000 g/mol] as initiators in the ROP of CL.	5
	**Table S2.** Triblock copolymers (PCL-*b*-$PTHF_x$-*b*-PCL) prepared using polytetrahydrofuran (PTHF) of different molecular weight [M_n= 250, 650, and 1000 g/mol] as initiators in the ROP of CL.	6
	**Table S3.** Triblock copolymers (PCL-*b*-PPG_x-*b*-PCL) prepared using polypropylene glycol (PPG) of different molecular weight [M_n= 425, 725, and 1000 g/mol] as initiators in the ROP of CL.	7
	**Table S4.** Thermal properties of triblock copolymers (PCL-*b*-PEG_x-*b*-PCL) prepared using polyethylene glycol (PEG) of different molecular weight [M_n= 200, 400, and 1000 g/mol] as initiators in the ROP of CL.	8
	**Table S5.** Thermal properties of triblock copolymers (PCL-*b*-$PTHF_x$-*b*-PCL) prepared using polytetrahydrofuran (PTHF) of different molecular weight [M_n= 250, 650, and 1000 g/mol] as initiators in the ROP of CL.	9
	**Table S6.** Thermal properties of triblock copolymers (PCL-*b*-PPG_x-*b*-PCL) prepared using polypropylene glycol (PPG) of different molecular weight [M_n= 425, 725, and 1000 g/mol] as initiators in the ROP of CL.	10
	**Table S7.** Comparison melting temperatures reported in previous studies on PCL-B-PCL triblock copolymers and some of the copolymers of the present study.	11
**Figures**		
	**Fig. S1** GPC curves of triblock copolymers: (a) PCL-*b*-PEG_{200}-*b*-PCL with four different DP, and (b) PCL-*b*-PPG-*b*-PCL with different length of segment B and DP=10.	12
	**Fig. S2** GPC chromatogram including PS calibration.	13
	**Fig. S3** Relation of PCL block length with ether content (%) and enthalpy (ΔH_m) of ABA triblock copolymers PCL-*b*-PEG_{1000}-*b*-PCL, PCL-*b*-$PTHF_{1000}$-*b*-PCL, and PCL-*b*-PPG_{1000}-*b*-PCL. For ether content, filled figures (Ether (%):▲■●) and for ΔH_{mPCL}, blue open figures (ΔH_{mPCL}: △□○).	14
	**Fig. S4** DSC thermograms **(a)** first heating [PCL-*b*-PEG_{200}-*b*-PCL_{10}, (T_{m1}= 33 °C, ΔH_{m1}= 18 J/g, T_{m2}= 46 °C, ΔH_{m2}= 34 J/g), PCL-*b*-$PTHF_{250}$-*b*-PCL_{10}, (T_{m1}= 33 °C, ΔH_{m1}= 19 J/g, T_{m2}= 46 °C, ΔH_{m2}= 41 J/g), and PCL-*b*-PPG_{425}-*b*-PCL_{10}, (T_{m1}= 36 °C, T_{m2}= 46 °C, ΔH_m= 60 J/g)], and **(b)** cooling [PCL-*b*-PEG_{200}-*b*-PCL_{10}, (T_c= 15 °C), PCL-*b*-$PTHF_{250}$-*b*-PCL_{10}, (T_c= 18 °C), and PCL-*b*-PPG_{425}-*b*-PCL_{10}, (T_c= 11 °C)].	15
	**Fig. S5** Effect of length of segment B in ABA triblock copolymers (a) Crystallinity (x_i) and (b) Melting temperature (T_m). Segment B: PEG (200, 400, 1000 g/mol), PTHF (250, 650, 1000 g/mol) y PPG (425, 725, 1000 g/mol). DP_{PCL} = 5.	16
	**Fig. S6** Effect of length of segment B in ABA triblock copolymers (a) Crystallinity (x_i) and (b) Melting temperature (T_m). Segment B: PEG (200,	17

400, 1000 g/mol), PTHF (250, 650, 1000 g/mol) y PPG (425, 725, 1000 g/mol). DP_{PCL} =15.

Fig. S7 Effect of length of segment B in ABA triblock copolymers (a) Crystallinity (x_i) and (b) Melting temperature (T_m). Segment B: PEG (200, 400, 1000 g/mol), PTHF (250, 650, 1000 g/mol) y PPG (425, 725, 1000 g/mol). DP_{PCL} = 20. 18

Fig. S8 FT-IR spectra of (a) PCL-b-PEG_{200}-b-PCL_{10} and (b) PU-1_{PEG} 19

Fig. S9 FT-IR spectra of (a) PCL-b-$PTHF_{250}$-b-PCL_{10} and (b) PU-2_{PTHF} 20

Fig. S10 FT-IR spectra of (a) PCL-b-PPG_{425}-b-PCL_{10} and (b) PU-3_{PPG} 21

Fig. S11 ^{1}H NMR (400 MHz) spectrum in $CDCl_3$ at room temperature for PU-3_{PPG} 22

Fig. S12 ^{13}C NMR (100 MHz) spectra in $CDCl_3$ at room temperature for: (a,c) PCL-*b*-$PTHF_{250}$-*b*-PCL_{10} and (b,d) PU-2_{PTHF}. 23

Data characterization

For PCL-*b*-PTHF$_{250}$-*b*-PCL (DP = 10).

M_n (calcd) = 1 390, M_n (NMR) = 1 560 (Conv. = 97%), M_n (GPC) = 3 273, M_w/M_n = 1.23, M_n (MALDI) = 1 400. IR (cm^{-1}) 3432 (ν, OH, PCL), 2937 (ν_{as}, CH_2, PCL), 2860 (ν_s, CH_2, PCL), 1723 (ν, C=O, PCL), 1472 (δ_s, CH_2, PCL), 1161 (ν_{as}, C–(C=O)–O, PCL), 1041 (ν_{as}, O–C–C, PCL), 734 (*ρ*, CH_2, PCL). NMR data for PCL-*b*-PTHF$_{250}$-*b*-PCL (DP = 10). ^{1}H NMR after derivatization with TFAA (500 MHz, $CDCl_3$, ppm): *δ* 4.49 [F_3C–CO–O–$\underline{CH_2}$–CH_2–CH_2–CH_2–O–, PTHF monosubstitution and unreacted PTHF], 4.34 [–CO–CH_2–CH_2–CH_2–CH_2–$\underline{CH_2}$–O–CO–CF_3, PCL], 4.26 [–CO–O–$\underline{CH_2}$–CH_2–CH_2–CH_2–O–CH_2–CH_2–CH_2–$\underline{CH_2}$–O–CO–, PTHF bisubstitution and F_3C–CO–O–CH_2–CH_2–CH_2–CH_2–O–CH_2–CH_2–CH_2–$\underline{CH_2}$–O–CO–, PTHF monosubstitution], 4.12 [(–CO–CH_2–CH_2–CH_2–CH_2–$\underline{CH_2}$–O–)$_n$, PCL], 3.95 [F_3C–CO–O–CH_2–CH_2–CH_2–$\underline{CH_2}$–O, PTHF monosubstitution and unreacted PTHF], 3.61 [–CO–O–CH_2–CH_2–CH_2–$\underline{CH_2}$–O–$\underline{CH_2}$–CH_2–CH_2–CH_2–O–CO– PTHF bisubstitution and F_3C–CO–O–CH_2–CH_2–CH_2–CH_2–O–$\underline{CH_2}$–CH_2 CH_2–CH_2–O–CO–, PTHF monosubstitution], 2.38 [(–CO–$\underline{CH_2}$–CH_2–CH_2–CH_2–CH_2–O–)$_n$, PCL], 1.77 [–CO–CH_2–$\underline{CH_2}$–CH_2–$\underline{CH_2}$–CH_2–O–CO–CF_3, PCL], 1.67 [(–CO–CH_2–$\underline{CH_2}$–CH_2–$\underline{CH_2}$–CH_2–O–)$_n$, PCL], 1.40 [(–CO–CH_2–CH_2–$\underline{CH_2}$–CH_2–CH_2–O–)$_n$, PCL].

For PCL-*b*-PPG$_{425}$-*b*-PCL (DP = 10).

M_n (calcd) = 1 570, M_n (NMR) = 2 020 (Conv. = 96%), M_n (GPC) = 4 102, M_w/M_n = 1.16. IR (cm^{-1}) 3437 (ν, OH, PCL), 2935 (ν_{as}, CH_2, PCL), 2863 (ν_s, CH_2, PCL), 1722 (ν, C=O, PCL), 1470 (δ_s, CH_2, PCL), 1161 (ν_{as}, C–(C=O)–O, PCL), 1042 (ν_{as}, O–C–C, PCL), 731 (*ρ*, CH_2, PCL). NMR data for PCL-*b*-PPG$_{425}$-*b*-PCL (DP = 10). ^{1}H NMR after derivatization with TFAA (500 MHz, $CDCl_3$, ppm): *δ* 5.09 [F_3C–CO–O–CH_2–$CH(CH_3)$–O–CH_2–$CH(CH_3)$–O–CH_2–$\underline{CH}(CH_3)$–O–CO–, PPG monosubstitution and –CO–O–CH_2–$\underline{CH}(CH_3)$–O–CH_2–$CH(CH_3)$–O–CH_2–$\underline{CH}(CH_3)$–O–CO–, PPG bisubstitution], 4.34 [–CO–CH_2–CH_2–CH_2–CH_2–$\underline{CH_2}$–O–CO–CF_3, PCL], 4.10 [(–CO–CH_2–CH_2–CH_2–CH_2–$\underline{CH_2}$–O–)$_n$, PCL], 4.09 [F_3C–CO–O–$\underline{CH_2}$–$\underline{CH}(CH_3)$–O–CH_2–$CH(CH_3)$–O–CO–, PPG monosubstitution], 3.72 [F_3C–CO–O–CH_2–$CH(CH_3)$–O–CH_2–$CH(CH_3)$–O–$\underline{CH_2}$–$CH(CH_3)$–O–CO–, PPG monosubstitution and –CO–O–$\underline{CH_2}$–$CH(CH_3)$–O–CH_2–$CH(CH_3)$–O–$\underline{CH_2}$–$CH(CH_3)$–O–CO–, PPG bisubstitution], 3.62 [(–$\underline{CH_2}$–$CH(CH_3)$–O–)$_x$, PPG monosubstitution and bisubstitution], 3.45 [(–CH_2–$\underline{CH}(CH_3)$–O–)$_x$, PPG monosubstitution and bisubstitution], 2.34 [(–CO–$\underline{CH_2}$–CH_2–CH_2–CH_2–CH_2–O–)$_n$, PCL], 1.77 [–CO–CH_2–$\underline{CH_2}$–CH_2–$\underline{CH_2}$–CH_2–O–CO–CF_3, PCL], 1.66 [(–CO–CH_2–$\underline{CH_2}$–CH_2–$\underline{CH_2}$–CH_2–O–)$_n$, PCL], 1.39 [(–CO–CH_2–CH_2–$\underline{CH_2}$–CH_2–CH_2–O–)$_n$, PCL], 1.33 [–CO–O–CH_2–$CH(\underline{CH_3})$–O–CH_2–$CH(\underline{CH_3})$–O–CO–, PPG bisubstitution and PPG monosubstitution].

Table S1. Triblock copolymers (PCL-*b*-PEG$_x$-*b*-PCL) prepared using polyethylene glycol (PEG) of different molecular weight [M_n= 200, 400, and 1000 g/mol] as initiators in the ROP of CL.

Sample	Initiator	Ether(%)[a,b]	DP(calcd)[c]	DP(NMR)[b,d]	M_n(calcd)[e]	M_n(NMR)[b,f]	M_n(GPC)[g]	M_n (calcd)/ M_n (GPC)	M_w/M_n[g]	Conv(%)
**PEG$_{200}$**		-	-	4.5	200	214				
PCL-*b*-PEG$_{200}$-*b*-PCL$_5$	PEG$_{200}$	28	5	4.9	780	770	1410	0.55	1.18	92
PCL-*b*-PEG$_{200}$-*b*-PCL$_{10}$	PEG$_{200}$	14	10	11.5	1350	1530	3145	0.42	1.24	95
PCL-*b*-PEG$_{200}$-*b*-PCL$_{15}$	PEG$_{200}$	11	15	14.3	1990	1850	3988	0.49	1.22	98
PCL-*b*-PEG$_{200}$-*b*-PCL$_{20}$	PEG$_{200}$	8	20	21.0	2440	2610	5628	0.43	1.24	98
**PEG$_{400}$**		-	-	8.9	400	408				
PCL-*b*-PEG$_{400}$-*b*-PCL$_5$	PEG$_{400}$	51	5	3.4	980	800	1419	0.69	1.17	89
PCL-*b*-PEG$_{400}$-*b*-PCL$_{10}$	PEG$_{400}$	29	10	11.2	1550	1680	2969	0.52	1.14	98
PCL-*b*-PEG$_{400}$-*b*-PCL$_{15}$	PEG$_{400}$	19	15	14.7	2110	2080	3939	0.53	1.13	99
PCL-*b*-PEG$_{400}$-*b*-PCL$_{20}$	PEG$_{400}$	15	20	19.8	2670	2660	5367	0.49	1.19	98
**PEG$_{1000}$**		-	-	22.0	1000	988				
PCL-*b*-PEG$_{1000}$-*b*-PCL$_5$	PEG$_{1000}$	67	5	4.3	1570	1470	1668	0.94	1.09	83
PCL-*b*-PEG$_{1000}$-*b*-PCL$_{10}$	PEG$_{1000}$	49	10	8.9	2120	2000	3431	0.61	1.13	98
PCL-*b*-PEG$_{1000}$-*b*-PCL$_{15}$	PEG$_{1000}$	41	15	13.4	2710	2410	4429	0.61	1.15	98
PCL-*b*-PEG$_{1000}$-*b*-PCL$_{20}$	PEG$_{1000}$	35	20	18.2	3120	2830	4871	0.64	1.26	97

[a]Obtained from the equation % PEG = ($MW_{initiator}/M_n$(NMR)) × 100; where $MW_{initiator}$ is the molecular weight of initiator (HOPEGOH).
[b]Determined by ^{1}H NMR in $CDCl_3$
[c]Obtained from CL/HOPEGOH feed molar ratio
[d]Using end-group analysis by ^{1}H NMR
[e]Obtained from the equation M_n(calcd) = (MW(CL))•(mmol CL/mmol HOPEGOH)+MW(OHPEGOH), where MW is the molecular weight of ε-caprolactone (CL, 114 g/mol) monomer or initiator (HOPEGOH)
[f]Obtained from the equation M_n(NMR) = (DP(NMR) × MW(repetitive unit))+MW(HOPEGOH), where MW is the molecular weight of the repetitive unit (114 g/mol) or initiator (HOPEGOH)
[g]Determined by gel permeation chromatography (GPC) using polystyrene standards.

Table S2. Triblock copolymers (PCL-*b*-$PTHF_x$-*b*-PCL) prepared using polytetrahydrofuran (PTHF) of different molecular weight [M_n= 250, 650, and 1000 g/mol] as initiators in the ROP of CL.

Sample	Initiator	Ether(%)[a,b]	DP(calcd)[c]	DP(NMR)[b,d]	M_n(calcd)[e]	M_n(NMR)[b,f]	M_n(GPC)[g]	M_n (calcd)/ M_n (GPC)	M_w/M_n[g]	Conv(%)
**$PTHF_{250}$**		-	-	3.3	250	254				
PCL-*b*-$PTHF_{250}$-*b*-PCL_5	$PTHF_{250}$	28	5	5.6	820	890	1601	0.51	1.19	96
PCL-*b*-$PTHF_{250}$-*b*-PCL_{10}	$PTHF_{250}$	16	10	11.4	1390	1560	3273	0.42	1.23	97
PCL-*b*-$PTHF_{250}$-*b*-PCL_{15}	$PTHF_{250}$	12	15	16.2	1960	2100	4853	0.40	1.16	99
PCL-*b*-$PTHF_{250}$-*b*-PCL_{20}	$PTHF_{250}$	10	20	20.3	2530	2570	5483	0.46	1.40	99
**$PTHF_{650}$**		-	-	8.7	650	644				
PCL-*b*-$PTHF_{650}$-*b*-PCL_5	$PTHF_{650}$	53	5	5.0	1220	1210	2512	0.48	1.28	96
PCL-*b*-$PTHF_{650}$-*b*-PCL_{10}	$PTHF_{650}$	35	10	10.6	1790	1850	3799	0.47	1.30	98
PCL-*b*-$PTHF_{650}$-*b*-PCL_{15}	$PTHF_{650}$	27	15	15.2	2360	2380	4825	0.49	1.17	99
PCL-*b*-$PTHF_{650}$-*b*-PCL_{20}	$PTHF_{650}$	22	20	19.4	2930	2850	5862	0.50	1.36	99
**$PTHF_{1000}$**		-	-	13.5	1000	989				
PCL-*b*-$PTHF_{1000}$-*b*-PCL_5	$PTHF_{1000}$	63	5	5.1	1580	1570	3002	0.52	1.35	96
PCL-*b*-$PTHF_{1000}$-*b*-PCL_{10}	$PTHF_{1000}$	46	10	10.2	2150	2150	4267	0.50	1.34	94
PCL-*b*-$PTHF_{1000}$-*b*-PCL_{15}	$PTHF_{1000}$	38	15	13.4	2720	2590	4789	0.56	1.40	98
PCL-*b*-$PTHF_{1000}$-*b*-PCL_{20}	$PTHF_{1000}$	33	20	17.4	3290	2970	6279	0.52	1.39	98

[a]Obtained from the equation % PTHF = ($MW_{initiator}/M_n$(NMR)) × 100; where $MW_{initiator}$ is the molecular weight of initiator (HOPTHFOH).
[b]Determined by ^1H NMR in $CDCl_3$
[c]Obtained from CL/HOPTHFOH feed molar ratio
[d]Using end-group analysis by ^1H NMR
[e]Obtained from the equation M_n(calcd) = (MW(CL))•(mmol CL/mmol HOPTHFOH)+MW(HOPTHFOH), where MW is the molecular weight of ε-caprolactone (CL, 114 g/mol) monomer or initiator (HOPTHFOH)
[f]Obtained from the equation M_n(NMR) = (DP(NMR) × MW(repetitive unit))+MW(HOPTHFOH), where MW is the molecular weight of the repetitive unit (114 g/mol) or initiator (HOPTHFOH)
[g]Determined by gel permeation chromatography (GPC) using polystyrene standards.

Table S3. Triblock copolymers (PCL-*b*-PPG$_x$-*b*-PCL) prepared using polypropylene glycol (PPG) of different molecular weight [M_n= 425, 725, and 1000 g/mol] as initiators in the ROP of CL.

Sample	Initiator	Ether(%)[a,b]	DP(calcd)[c]	DP(NMR)[b,d]	M_n(calcd)[e]	M_n(NMR)[b,f]	M_n(GPC)[g]	M_n (calcd)/ M_n (GPC)	M_w/M_n[g]	Conv(%)
**PPG$_{425}$**			-	6.8	425	396				-
PCL-*b*-PPG$_{425}$-*b*-PCL$_5$	PPG$_{425}$	43	5	7.8	1000	1280	2373	0.42	1.15	89
PCL-*b*-PPG$_{425}$-*b*-PCL$_{10}$	PPG$_{425}$	27	10	14.2	1570	2020	4102	0.38	1.16	96
PCL-*b*-PPG$_{425}$-*b*-PCL$_{15}$	PPG$_{425}$	20	15	18.7	2130	2530	6079	0.35	1.14	98
PCL-*b*-PPG$_{425}$-*b*-PCL$_{20}$	PPG$_{425}$	16	20	22.7	2680	2980	5719	0.46	1.48	98
**PPG$_{725}$**			-	12.1	720	705				-
PCL-*b*-PPG$_{725}$-*b*-PCL$_5$	PPG$_{725}$	56	5	8.4	1290	1660	2623	0.49	1.40	90
PCL-*b*-PPG$_{725}$-*b*-PCL$_{10}$	PPG$_{725}$	39	10	13.9	1860	2290	3743	0.49	1.44	96
PCL-*b*-PPG$_{725}$-*b*-PCL$_{15}$	PPG$_{725}$	30	15	18.1	2410	2770	4799	0.50	1.53	97
PCL-*b*-PPG$_{725}$-*b*-PCL$_{20}$	PPG$_{725}$	24	20	24.2	3010	3460	6357	0.47	1.41	98
**PPG$_{1000}$**			-	17.0	1000	985				-
PCL-*b*-PPG$_{1000}$-*b*-PCL$_5$	PPG$_{1000}$	64	5	8.2	1570	1920	2685	0.58	1.24	90
PCL-*b*-PPG$_{1000}$-*b*-PCL$_{10}$	PPG$_{1000}$	47	10	13.6	2130	2530	3764	0.56	1.35	96
PCL-*b*-PPG$_{1000}$-*b*-PCL$_{15}$	PPG$_{1000}$	37	15	16.2	2700	2830	4585	0.58	1.35	98
PCL-*b*-PPG$_{1000}$-*b*-PCL$_{20}$	PPG$_{1000}$	30	20	22.5	3280	3550	5596	0.58	1.45	98

[a]Obtained from the equation % PPG = ($MW_{initiator}/M_n$(NMR)) × 100; where $MW_{initiator}$ is the molecular weight of initiator (HOPPGOH).
[b]Determined by ^{1}H NMR in $CDCl_3$
[c]Obtained from CL/HOPPGOH feed molar ratio
[d]Using end-group analysis by ^{1}H NMR
[e]Obtained from the equation M_n(calcd) = (MW(CL))•(mmol CL/mmol HOPPGOH)+MW(OHPPGOH), where MW is the molecular weight of ε-caprolactone (CL, 114 g/mol) monomer or initiator (HOPPGOH)
[f]Obtained from the equation M_n(NMR) = (DP(NMR) × MW(repetitive unit))+MW(HOPPGOH), where MW is the molecular weight of the repetitive unit (114 g/mol) or initiator (HOPPGOH)
[g]Determined by gel permeation chromatography (GPC) using polystyrene standards.

Table S4. Thermal properties of triblock copolymers (PCL-*b*-PEG_x-*b*-PCL) prepared using polyethylene glycol (PEG) of different molecular weight [M_n= 200, 400, and 1000 g/mol] as initiators in the ROP of CL.

Sample	Initiator	Ether(%)[a,b]	DP(calcd)[c]	DP(NMR)[b,d]	M_n(calcd)[e]	M_n(NMR)[b,f]	T_c(°C)[g]	ΔH_c (J/g)[g]	T_{mPCL}(°C)[g]	ΔH_m (J/g)[g]	x_i(%)[g,h]
PEG_{200}		-	-	4.5	200	214			-50[i]	-	-
PCL-*b*-PEG_{200}-*b*-PCL_5	PEG_{200}	28	5	4.9	780	770	-	-	6	0.7	0.5
PCL-*b*-PEG_{200}-*b*-PCL_{10}	PEG_{200}	14	10	11.5	1 350	1 530	15	50	32,41	50	37
PCL-*b*-PEG_{200}-*b*-PCL_{15}	PEG_{200}	11	15	14.3	1 990	1 850	20	73	35,42	76	56
PCL-*b*-PEG_{200}-*b*-PCL_{20}	PEG_{200}	8	20	21.0	2 440	2 610	23	75	43,48	77	57
PEG_{400}		-	-	8.9	400	408			1[j]	88[j]	-
PCL-*b*-PEG_{400}-*b*-PCL_5	PEG_{400}	51	5	3.4	980	800	-	-	2[j]	-	-
PCL-*b*-PEG_{400}-*b*-PCL_{10}	PEG_{400}	29	10	11.2	1 550	1 680	2	51	20,33	53	39
PCL-*b*-PEG_{400}-*b*-PCL_{15}	PEG_{400}	19	15	14.7	2 110	2 080	17	66	33,39	67	49
PCL-*b*-PEG_{400}-*b*-PCL_{20}	PEG_{400}	15	20	19.8	2 670	2 660	23	69	40,46	70	51
PEG_{1000}		-	-	22.0	1 000	988			34	139	-
PCL-*b*-PEG_{1000}-*b*-PCL_5	PEG_{1000}	67	5	4.3	1 570	1 470	-7	81	-7[j],21	37[j],65(15[k])	11
PCL-*b*-PEG_{1000}-*b*-PCL_{10}	PEG_{1000}	49	10	8.9	2 120	2 000	-6	59	17	60(30[k])	22
PCL-*b*-PEG_{1000}-*b*-PCL_{15}	PEG_{1000}	41	15	13.4	2 710	2 410	-4[j],14	5[j],46	16[j],30,37	56(33[k])	24
PCL-*b*-PEG_{1000}-*b*-PCL_{20}	PEG_{1000}	35	20	18.2	3 120	2 830	-8[j],20	4[j],49	18[j],36,43	3[j],42	31

[a]Obtained from the equation % PEG = ($MW_{initiator}$/M_n(NMR)) × 100; where $MW_{initiator}$ is the molecular weight of initiator (HOPEGOH).

[b]Determined by ^{1}H NMR in $CDCl_3$

[c]Obtained from CL/HOPEGOH feed molar ratio

[d]Using end-group analysis by ^{1}H NMR

[e]Obtained from the equation M_n(calcd) = (MW(CL))•(mmol CL/mmol HOPEGOH)+MW(HOPEGOH), where MW is the molecular weight of ε-caprolactone (CL, 114 g/mol) monomer or initiator (HOPEGOH)

[f]Obtained from the equation M_n(NMR) = (DP(NMR) × MW(repetitive unit))+MW(HOPEGOH), where MW is the molecular weight of the repetitive unit (114 g/mol) or initiator (HOPEGOH)

[g]Obtained by DSC analysis (second heating)

[h]Using the value of 135.3 J/g for a PCL 100% crystalline, the crystallinity of PCL (x_{PCL}) was calculated

[i]Reported value for PEG (M_n=200 g/mol), which is liquid a room temperature and with a melting point ≤ 0 °C

[j]Signal belonging to the melting or crystallization temperature of the PEG segment.

[k]Enthalpy of fusion attributed to the PCL, obtained by the equation ΔH_{mPC}L=(ΔH_m)•(weight fraction of PCL).

Table S5. Thermal properties of triblock copolymers (PCL-*b*-PTHF$_x$-*b*-PCL) prepared using polytetrahydrofuran (PTHF) of different molecular weight [M_n= 250, 650, and 1000 g/mol] as initiators in the ROP of CL.

Sample	Initiator	Ether(%)[a,b]	DP(calcd)[c]	DP(NMR)[b,d]	M_n(calcd)[e]	M_n(NMR)[b,f]	T_c(°C)[g]	ΔH_c (J/g)[g]	T_{mPCL}(°C)[g]	ΔH_m (J/g)[g]	x_i(%)[g,h]
PTHF$_{250}$		-	-	3.3	250	254			-16[i]	53	-
PCL-*b*-PTHF$_{250}$-*b*-PCL$_5$	PTHF$_{250}$	28	5	5.6	820	890	-18	42	-16[i],5,13	43(31[j])	23
PCL-*b*-PTHF$_{250}$-*b*-PCL$_{10}$	PTHF$_{250}$	16	10	11.4	1 390	1 560	18	61	33,41	63	46
PCL-*b*-PTHF$_{250}$-*b*-PCL$_{15}$	PTHF$_{250}$	12	15	16.2	1 960	2 100	18	70	39,41	74	54
PCL-*b*-PTHF$_{250}$-*b*-PCL$_{20}$	PTHF$_{250}$	10	20	20.3	2 530	2 570	22	65	41,47	72	53
PTHF$_{650}$		-	-	8.7	650	644			15[k]	71[i]	-
PCL-*b*- PTHF$_{650}$-*b*-PCL$_5$	PTHF$_{650}$	53	5	5.0	1 220	1 210	-18	62	0.1,11	60(28[j])	20
PCL-*b*- PTHF$_{650}$-*b*-PCL$_{10}$	PTHF$_{650}$	35	10	10.6	1 790	1 850	-10[i],9	11[i],46	7[i],27,35	11[i],43	31
PCL-*b*- PTHF$_{650}$-*b*-PCL$_{15}$	PTHF$_{650}$	27	15	15.2	2 360	2 380	14	63	32,38	64	47
PCL-*b*-PTHF$_{650}$-*b*-PCL$_{20}$	PTHF$_{650}$	22	20	19.4	2 930	2 850	19	64	40,46	65	48
PTHF$_{1000}$		-	-	13.5	1 000	989			10[i]	139	-
PCL-*b*-PTHF$_{1000}$-*b*-PCL$_5$	PTHF$_{1000}$	63	5	5.1	1 580	1 570	-9	72	10,17	69(25[j])	18
PCL-*b*-PTHF$_{1000}$-*b*-PCL$_{10}$	PTHF$_{1000}$	46	10	10.2	2 150	2 150	-1,7	56	14[i],26,36	19[i],26	19
PCL-*b*-PTHF$_{1000}$-*b*-PCL$_{15}$	PTHF$_{1000}$	38	15	13.4	2 720	2 590	-3[i],12	10[i], 50	13[i],33,42	11[i],48	35
PCL-*b*-PTHF$_{1000}$-*b*-PCL$_{20}$	PTHF$_{1000}$	33	20	17.4	3 290	2 970	-5[i],19	9[i],54	18[i],40,46	3[i],64	47

[a]Obtained from the equation % PTHF = ($MW_{initiator}$/M_n(NMR)) × 100; where $MW_{initiator}$ is the molecular weight of initiator (HOPTHFOH).

[b]Determined by ^{1}H NMR in $CDCl_3$

[c]Obtained from CL/HOPTHFOH feed molar ratio

[d]Using end-group analysis by ^{1}H NMR

[e]Obtained from the equation M_n(calcd) = (MW(CL))•(mmol CL/mmol HOPTHFOH)+MW(HOPTHFOH), where MW is the molecular weight of ε-caprolactone (CL, 114 g/mol) monomer or initiator (HOPTHFOH)

[f]Obtained from the equation M_n(NMR) = (DP(NMR) × MW(repetitive unit))+MW(HOPTHFOH), where MW is the molecular weight of the repetitive unit (114 g/mol) or initiator (HOPTHFOH)

[g]Obtained by DSC analysis (second heating)

[h]Using the value of 135.3 J/g for a PCL 100% crystalline, the crystallinity of PCL (x_{PCL}) was calculated

[i]Signal belonging to the melting temperature of the PTHF segment.

[j]Enthalpy of fusion attributed to the PCL, obtained by the equation ΔH_{mPC}L=(ΔH_m)•(weight fraction of PCL).

Table S6. Thermal properties of triblock copolymers (PCL-*b*-PPG_x-*b*-PCL) prepared using polypropylene glycol (PPG) of different molecular weight [M_n= 425, 725, and 1000 g/mol] as initiators in the ROP of CL.

Sample	Initiator	Ether(%)[a,b]	DP(calcd)[c]	DP(NMR)[b,d]	M_n(calcd)[e]	M_n(NMR)[b,f]	T_c(°C)[g]	ΔH_c (J/g)[g]	T_{mPCL}(°C)[g]	ΔH_m (J/g)[g]	x_i(%)[g,h]
PPG_{425}		-	-	6.8	425	396			-		
PCL-*b*-PPG_{425}-*b*-PCL_5	PPG_{425}	43	5	7.8	1 000	1 280	-15	37	2,20	33	24
PCL-*b*-PPG_{425}-*b*-PCL_{10}	PPG_{425}	27	10	14.2	1 570	2 020	11	57	28,39	56	41
PCL-*b*-PPG_{425}-*b*-PCL_{15}	PPG_{425}	20	15	18.7	2 130	2 530	18	62	38,45	62	45
PCL-*b*-PPG_{425}-*b*-PCL_{20}	PPG_{425}	16	20	22.7	2 680	2 980	20	60	40,48	61	45
PPG_{725}		-	-	12.1	720	705			-		
PCL-*b*-PPG_{725}-*b*-PCL_5	PPG_{725}	56	5	8.4	1 290	1 660	-11	24	9,30	22	16
PCL-*b*-PPG_{725}-*b*-PCL_{10}	PPG_{725}	39	10	13.9	1 860	2 290	8	44	29,41	41	30
PCL-*b*-PPG_{725}-*b*-PCL_{15}	PPG_{725}	30	15	18.1	2 410	2 770	11	54	34,45	55	40
PCL-*b*-PPG_{725}-*b*-PCL_{20}	PPG_{725}	24	20	24.2	3 010	3 460	21	58	40,47	58	42
PPG_{1000}		-	-	17	1 000	985			-		
PCL-*b*-PPG_{1000}-*b*-PCL_5	PPG_{1000}	64	5	8.2	1 570	1 920	-18	5	-4,20	18	13
PCL-*b*-PPG_{1000}-*b*-PCL_{10}	PPG_{1000}	47	10	13.6	2 130	2 530	7	34	27,39	33	24
PCL-*b*-PPG_{1000}-*b*-PCL_{15}	PPG_{1000}	37	15	16.2	2 700	2 700	10	55	30,41	57	42
PCL-*b*-PPG_{1000}-*b*-PCL_{20}	PPG_{1000}	30	20	22.5	3 280	3 550	19	53	38,46	52	38

[a]Obtained from the equation % PPG = ($MW_{initiator}$/M_n(NMR)) × 100; where $MW_{initiator}$ is the molecular weight of initiator (HOPPGOH).
[b]Determined by ^1H NMR in $CDCl_3$
[c]Obtained from CL/HOPPGOH feed molar ratio
[d]Using end-group analysis by ^1H NMR
[e]Obtained from the equation M_n(calcd) = (MW(CL))•(mmol CL/mmol HOPPGOH)+MW(HOPPGOH), where MW is the molecular weight of ε-caprolactone (CL, 114 g/mol) monomer or macroinitiator (HOPPGOH)
[f]Obtained from the equation M_n(NMR) = (DP(NMR) × MW(repetitive unit))+MW(HOPPGOH), where MW is the molecular weight of the repetitive unit (114 g/mol) or macroinitiator (HOPPGOH)
[g]Obtained by DSC analysis (second heating)
[h]Using the value of 135.3 J/g for a PCL 100% crystalline, the crystallinity of PCL (x_i) was calculated.

Table S7. Comparison melting temperatures reported in previous studies on PCL-B-PCL triblock copolymers and some of the copolymers of the present study.

Chemical specie	M_n of chemical specie (g/mol)	DP PCL	Reference	Thermal properties	
				T_m	Value (°C)
PCL-PEG$_{1000}$-PCL	2120	10	This work	Yes	17
PCL-PEG$_{1000}$-PCL	2710	15	This work	Yes	16*,30,37
PCL-PEG$_{1000}$-PCL	3120	20	This work	Yes	18*,36,43
PCL-PEG-PCL	4600	22	Piao, 2003 [12]	Yes	41, 48*
PCL-PEG-PCL	Over 8000	-	Zhang, 2016 [29]	No	
PCL-PEG-PCL	2000	8	Noormohammadi, 2021 [16]	Yes	25, 35
PCL-PEG-PCL	4450	25	Alami-milani, 2018 [24]	Yes	38, 57
PCL-PEG-PCL	Over 16000	120	Singh, 2020 [30]	Yes	59
PCL-PTHF$_{1000}$-PCL	2150	10	This work	Yes	14*,26,36
PCL-PTHF$_{1000}$-PCL	2590	15	This work	Yes	13*,33,42
PCL-PTHF$_{1000}$-PCL	2970	20	This work	Yes	18*,40,46
PCL-PTHF-PCL	2000	-	Ruedas-Larraz, 2009 [44]	No	
PCL-PTHF-PCL	2000	-	Jiang, 2018 [51]	No	
PCL-PTHF-PCL	2000	-	Mi, 2017 [43]	No	
PCL-PTHF-PCL	2000	8	Li, 2004 [48]	Yes	16, 26
PCL-PTHF-PCL	2000	-	do Patrocínio, 2019 [56]	Yes	56
PCL-PPG$_{1000}$-PCL	2530	10	This work	Yes	27,39
PCL-PPG$_{1000}$-PCL	2700	15	This work	Yes	30,41
PCL-PPG$_{1000}$-PCL	3550	20	This work	Yes	38,46
PCL-PPG-PCL	2000	8	Shi, 2025 [33]	Yes	17
PCL-PPG-PCL	1000	8	Lee, 2011 [36]	No	

*T_m attributed to the polyether segment.

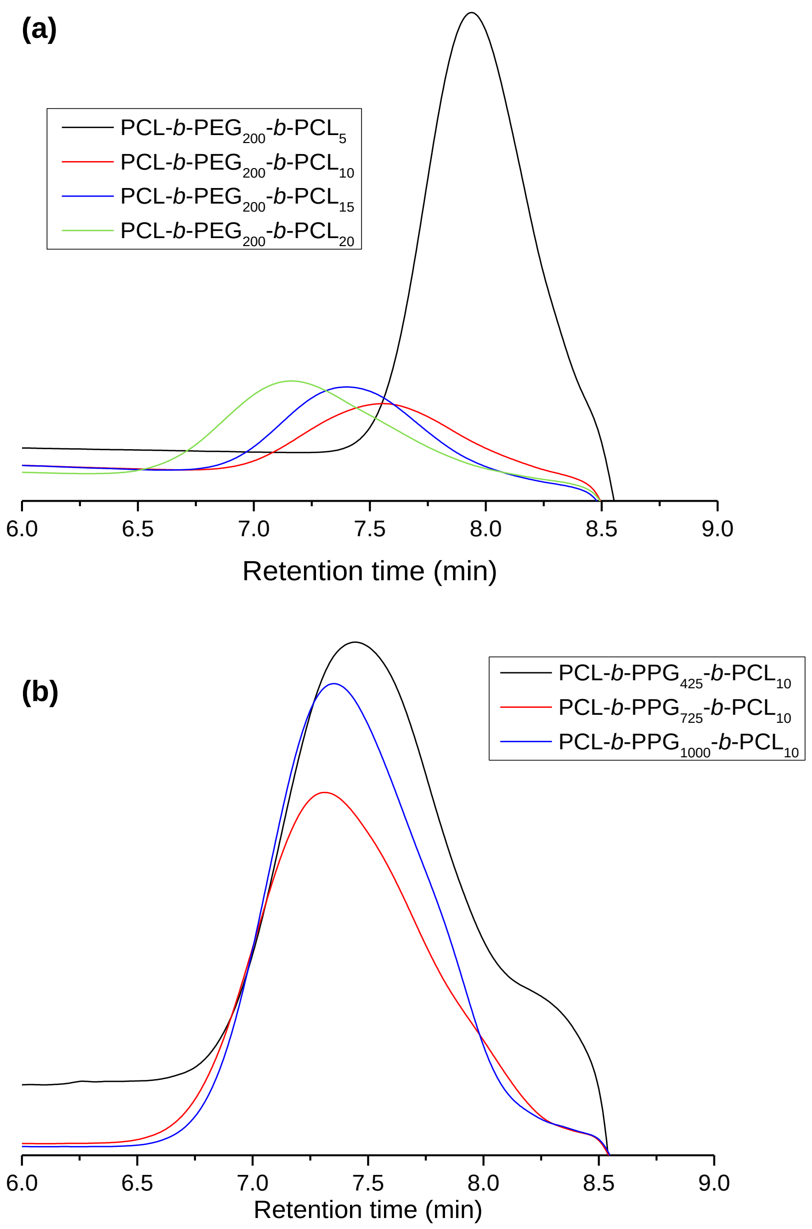

Fig. S1 GPC curves of triblock copolymers: (a) PCL-*b*-PEG_{200}-*b*-PCL with four different DP, and (b) PCL-*b*-PPG-*b*-PCL with different length of segment B and DP=10.

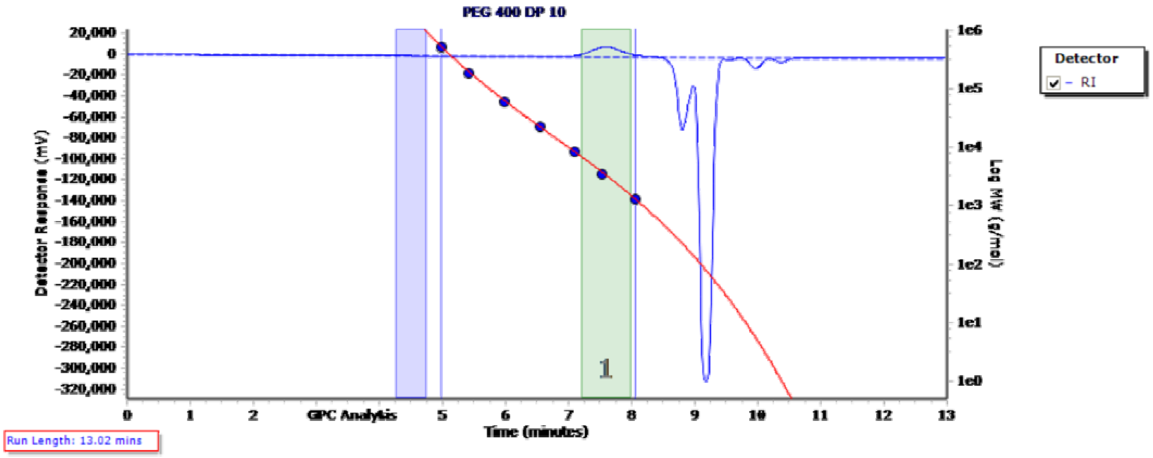

Fig. S2 GPC chromatogram including PS calibration.

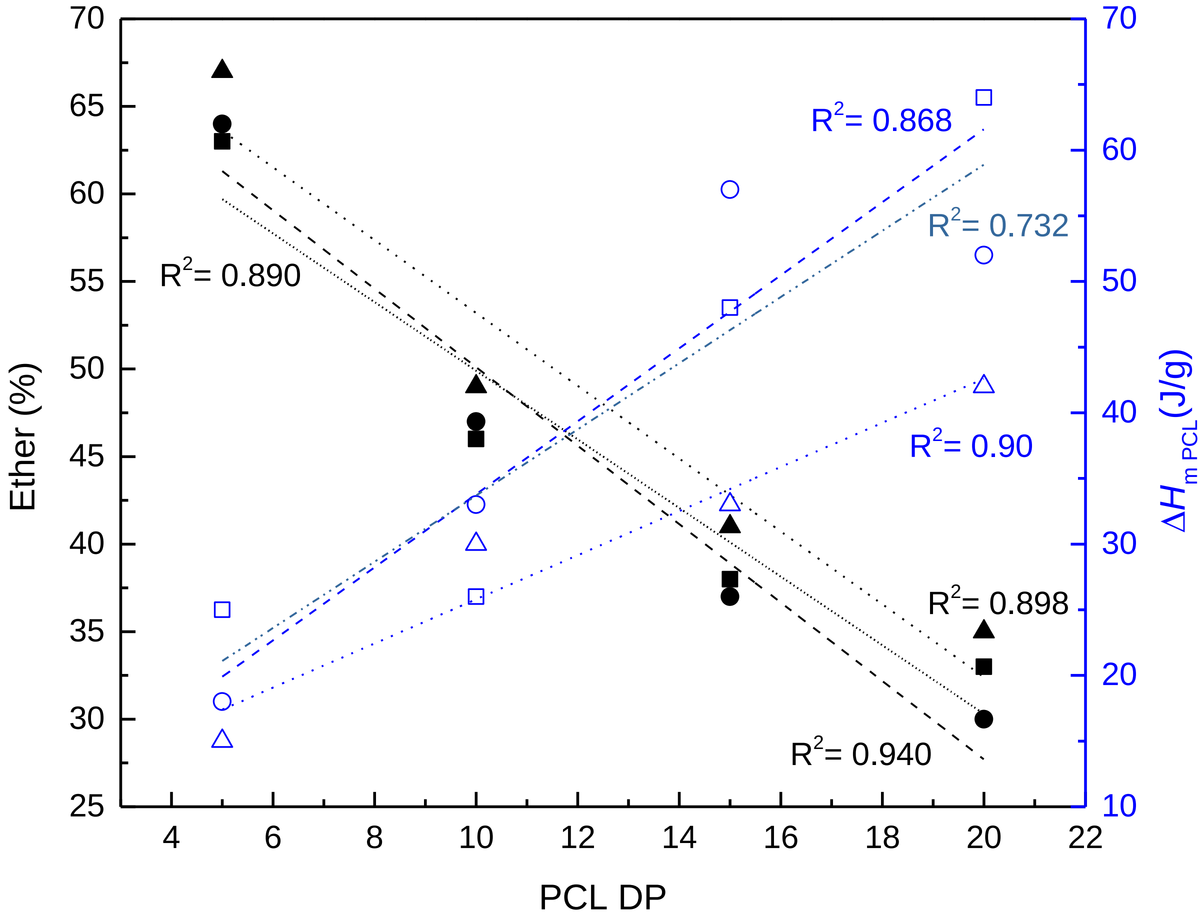

Fig. S3 Relation of PCL block length with ether content (%) and enthalpy (ΔH_m) of ABA triblock copolymers PCL-*b*-PEG_{1000}-*b*-PCL, PCL-*b*-$PTHF_{1000}$-*b*-PCL, and PCL-*b*-PPG_{1000}-*b*-PCL. For ether content, filled figures (Ether (%): ▲■●) and for ΔH_{mPCL}, blue open figures (ΔH_{mPCL}: △□○).

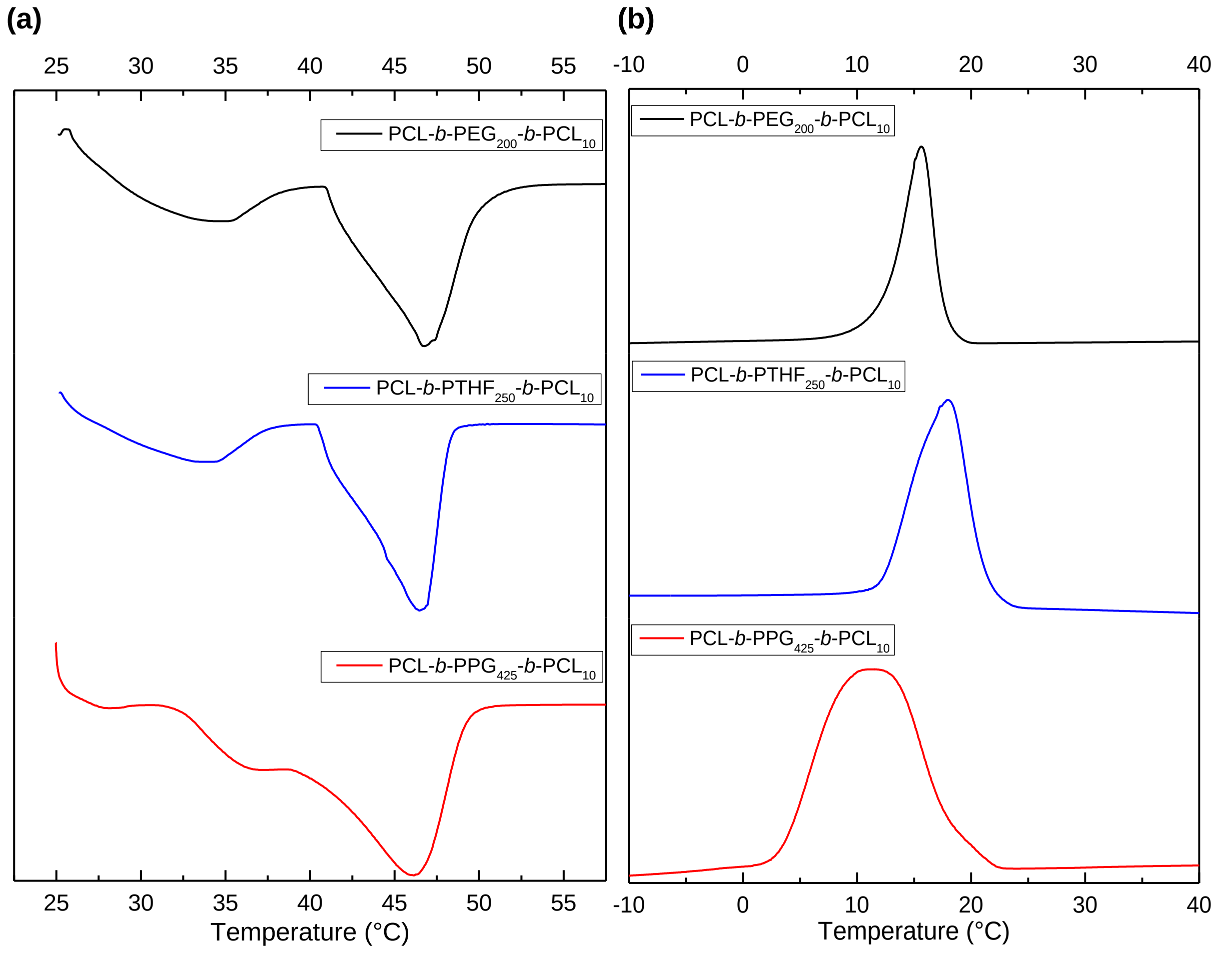

Fig. S4 DSC thermograms **(a)** first heating [PCL-*b*-PEG_{200}-*b*-PCL_{10}, (T_{m1}= 33 °C, ΔH_{m1}= 18 J/g, T_{m2}= 46 °C, ΔH_{m2}= 34 J/g), PCL-*b*-$PTHF_{250}$-*b*-PCL_{10}, (T_{m1}= 33 °C, ΔH_{m1}= 19 J/g, T_{m2}= 46 °C, ΔH_{m2}= 41 J/g), and PCL-*b*-PPG_{425}-*b*-PCL_{10}, (T_{m1}= 36 °C, T_{m2}= 46 °C, ΔH_{m}= 60 J/g)], and **(b)** cooling [PCL-*b*-PEG_{200}-*b*-PCL_{10}, (T_c= 15 °C), PCL-*b*-$PTHF_{250}$-*b*-PCL_{10}, (T_c= 18 °C), and PCL-*b*-PPG_{425}-*b*-PCL_{10}, (T_c= 11 °C)].

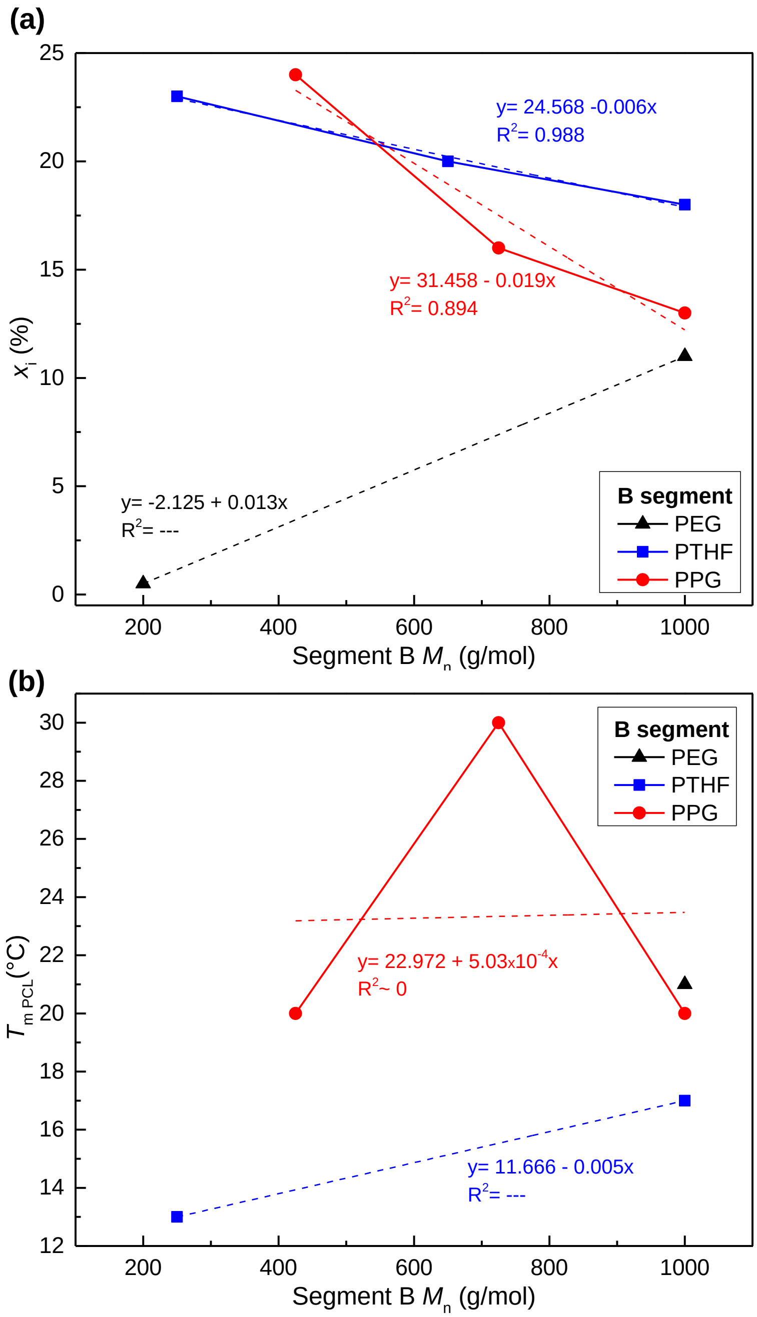

Fig. S5 Effect of length of segment B in ABA triblock copolymers (a) Crystallinity (x_i) and (b) Melting temperature (T_m). Segment B: PEG (200, 400, 1000 g/mol), PTHF (250, 650, 1000 g/mol) y PPG (425, 725, 1000 g/mol). DP_{PCL} = 5.

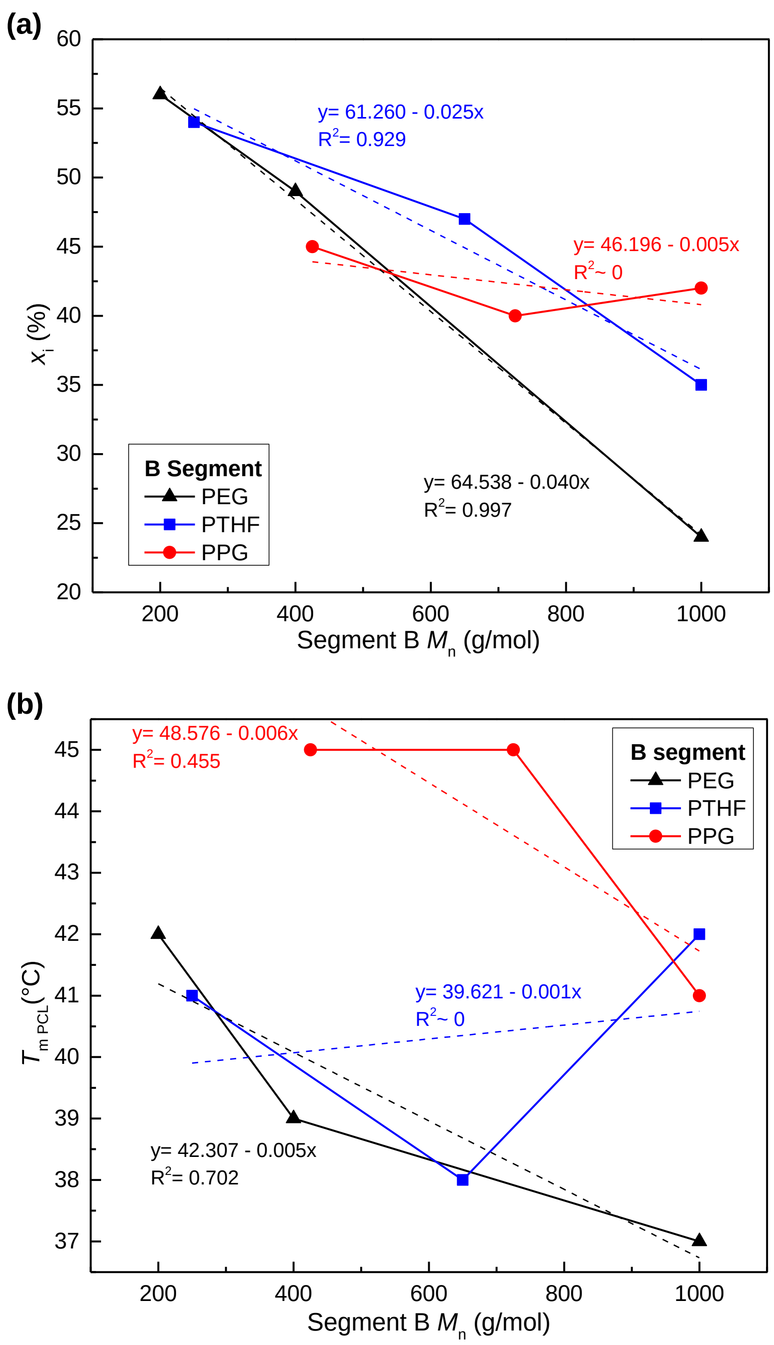

Fig. S6 Effect of length of segment B in ABA triblock copolymers (a) Crystallinity (x_i) and (b) Melting temperature (T_m). Segment B: PEG (200, 400, 1000 g/mol), PTHF (250, 650, 1000 g/mol) y PPG (425, 725, 1000 g/mol). $DP_{PCL} = 15$.

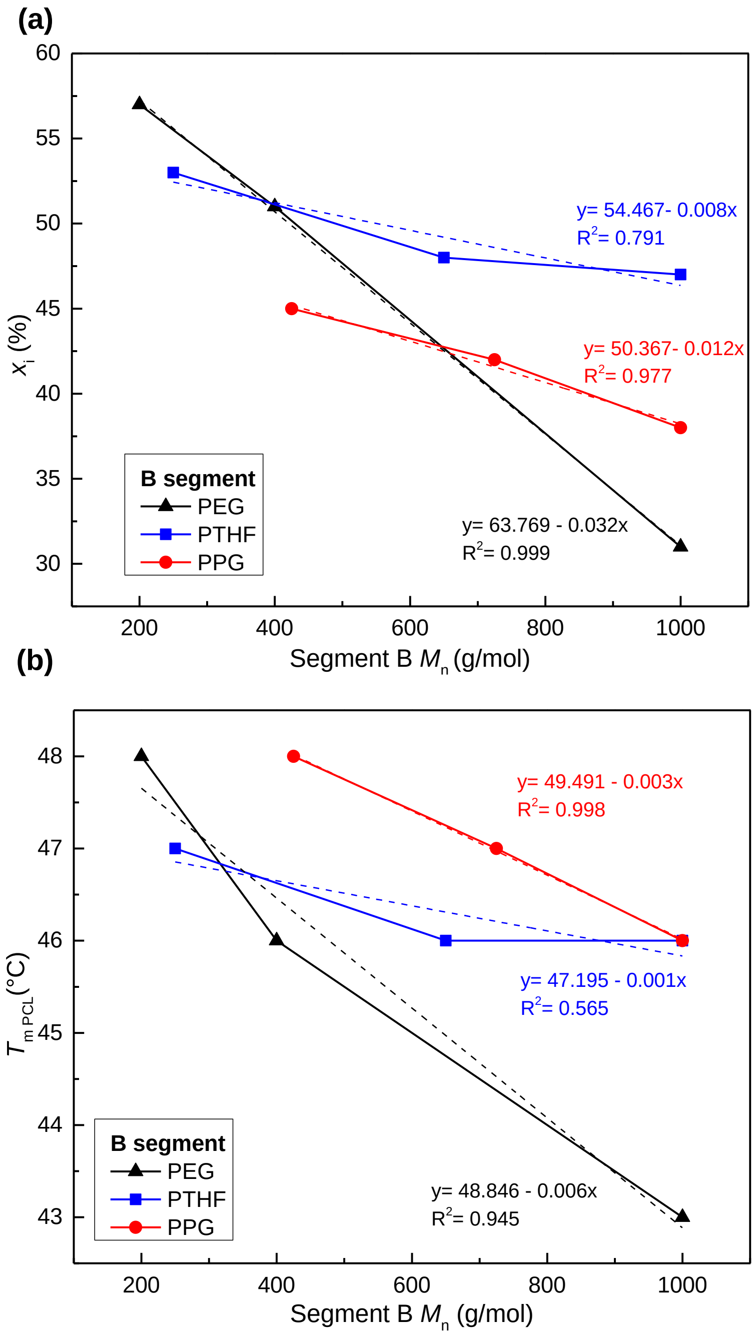

Fig. S7 Effect of length of segment B in ABA triblock copolymers (a) Crystallinity (x_i) and (b) Melting temperature (T_m). Segment B: PEG (200, 400, 1000 g/mol), PTHF (250, 650, 1000 g/mol) y PPG (425, 725, 1000 g/mol). DP_{PCL} = 20.

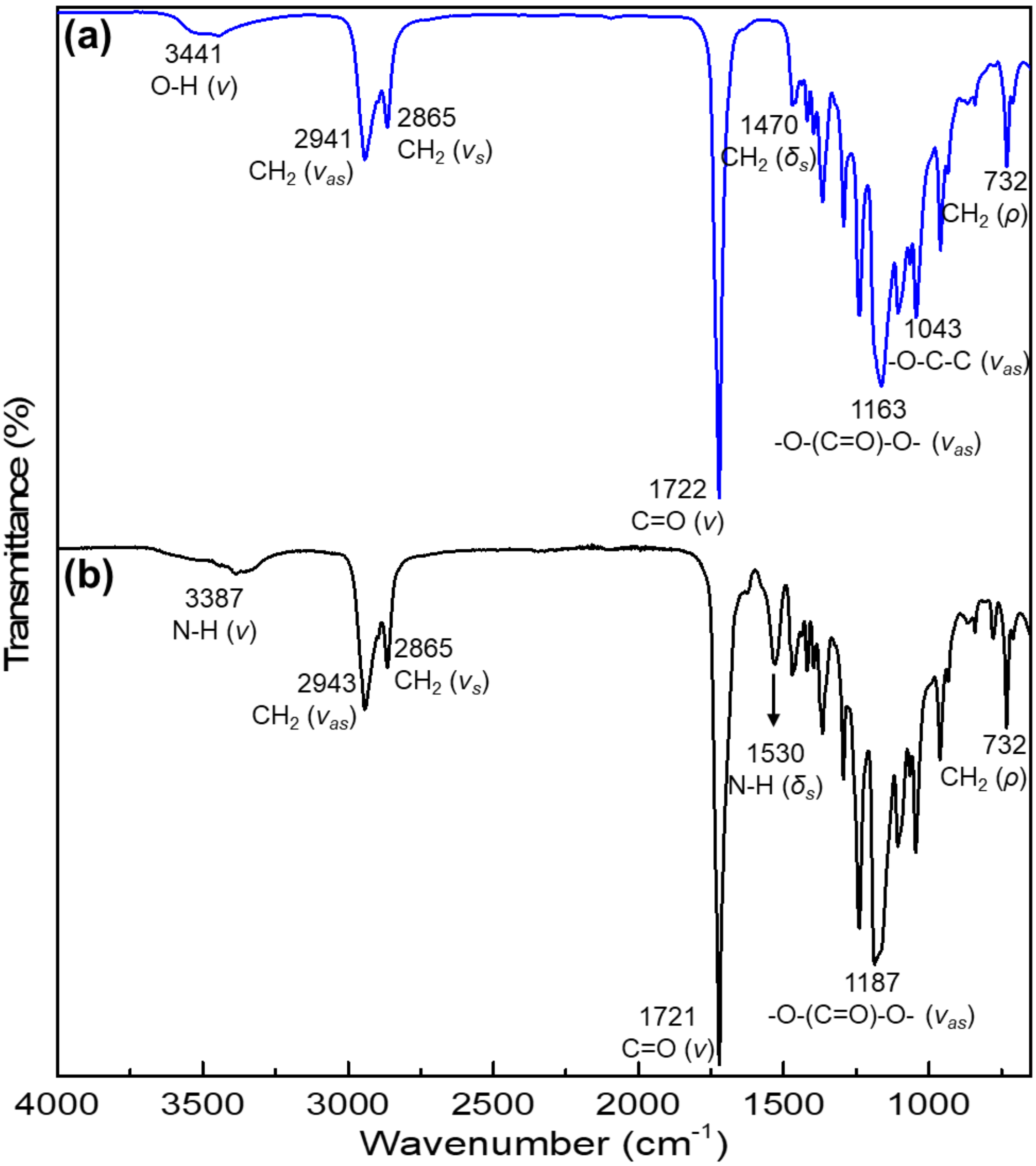

Fig. S8 FT-IR spectra of (a) PCL-b-PEG_{200}-b-PCL_{10} and (b) PEU-1_{PEG}

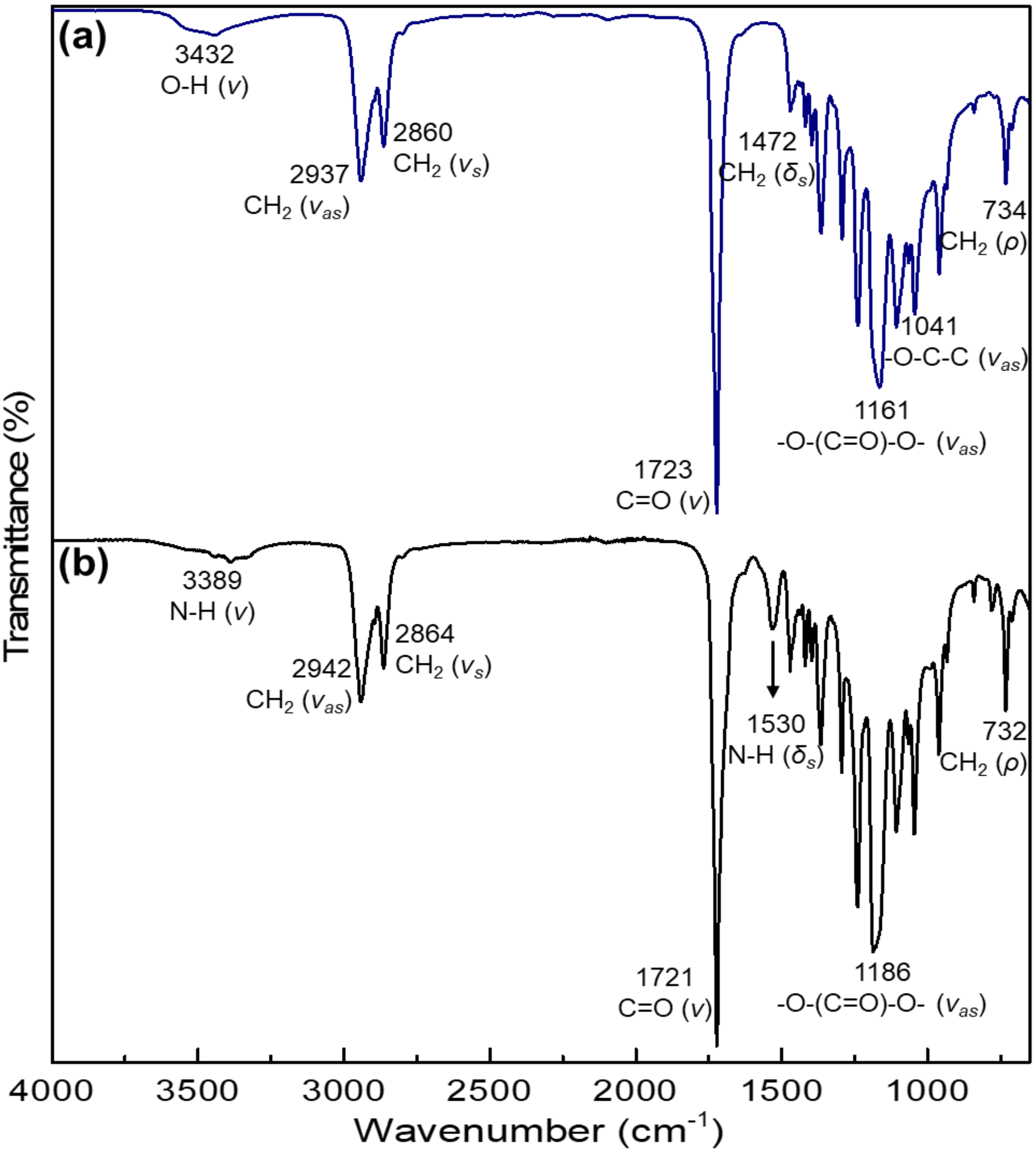

Fig. S9 FT-IR spectra of (a) PCL-b-PTHF$_{250}$-b-PCL$_{10}$ and (b) PEU-2$_{PTHF}$

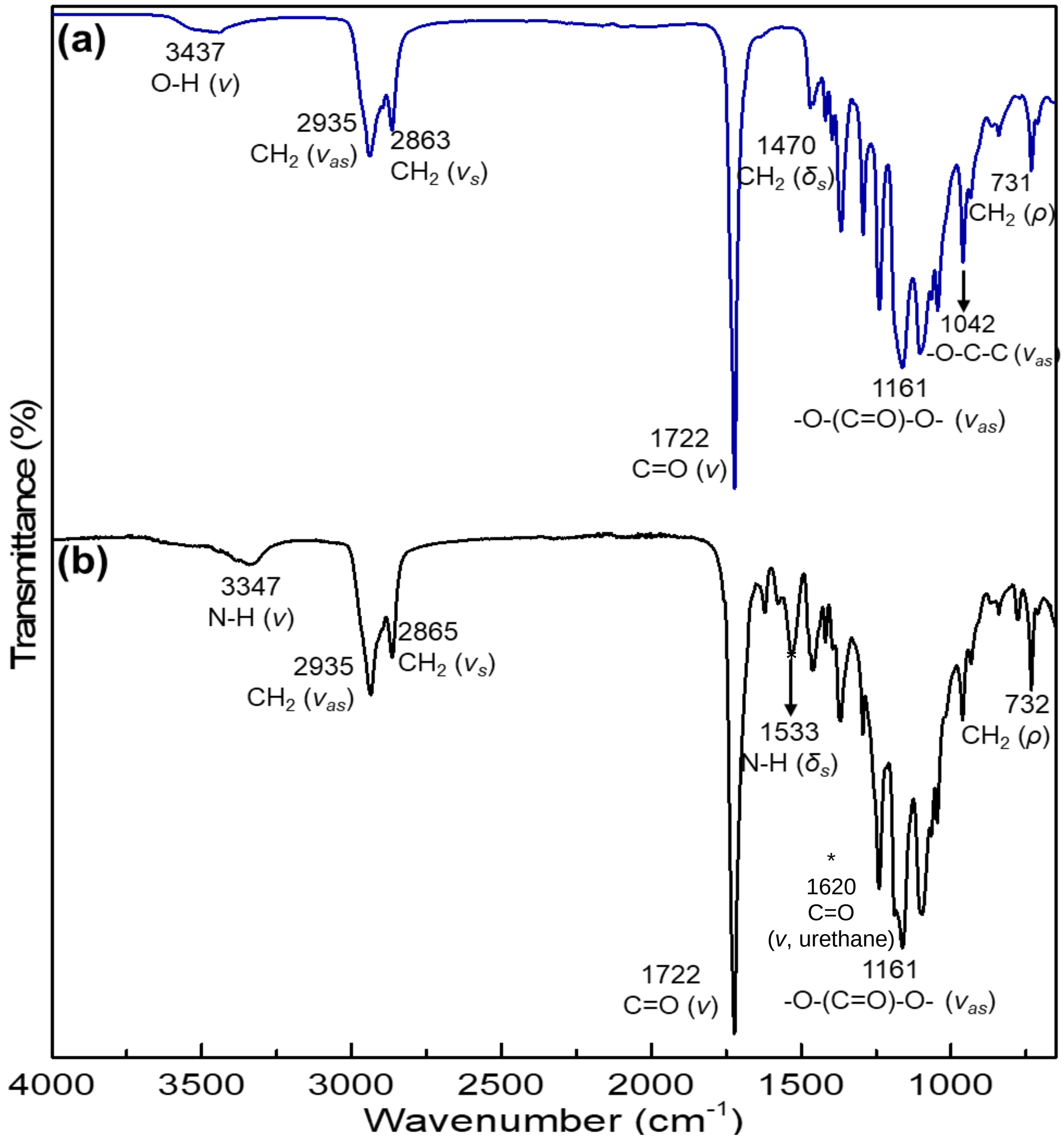

Fig. S10 FT-IR spectra of (a) PCL-b-PPG_{425}-b-PCL_{10} and (b) PEU-3_{PPG}

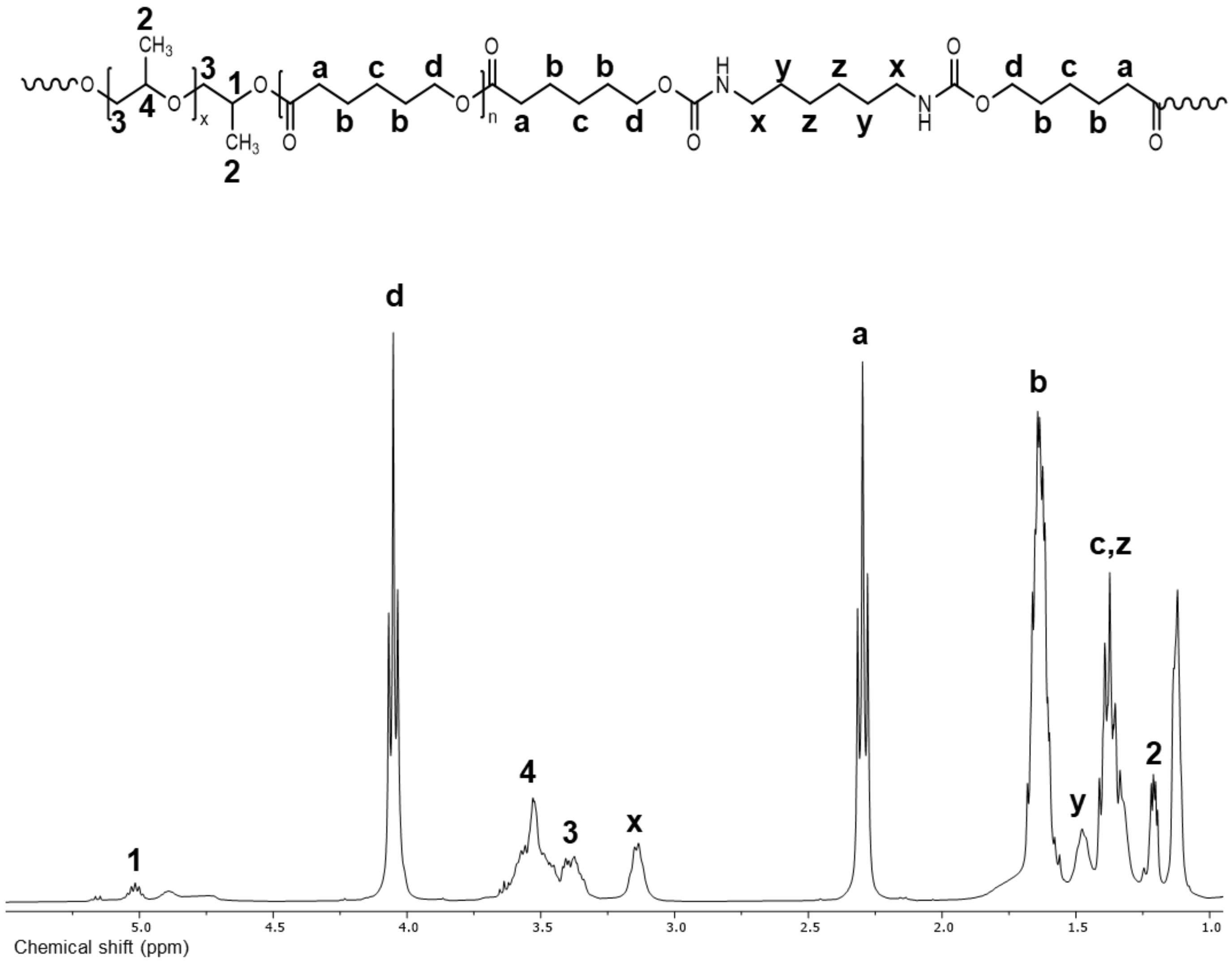

Fig. S11 ^{1}H NMR (400 MHz) spectrum in $CDCl_3$ at room temperature for PEU-3_{PPG}

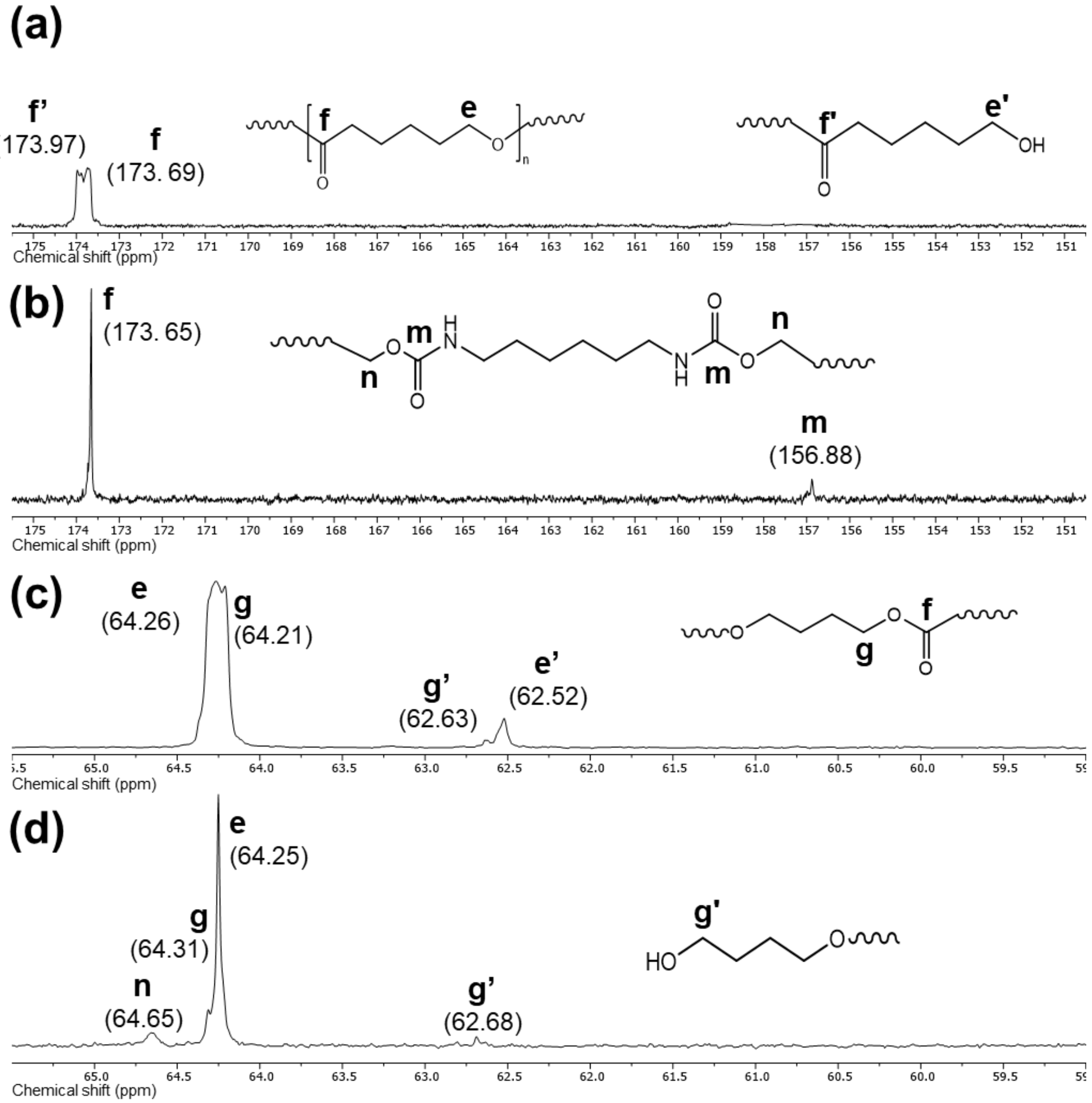

Fig. S12 ^{13}C NMR (100 MHz) spectra in $CDCl_3$ at room temperature for: (a,c) PCL-*b*-PTHF$_{250}$-*b*-PCL$_{10}$ and (b,d) PU-2$_{PTHF}$.
